# A Model for Aryl Hydrocarbon Receptor-Activated Gene Expression Shows Potency and Efficacy Changes and Predicts Squelching Due to Competition for Transcription Co-Activators

**DOI:** 10.1371/journal.pone.0127952

**Published:** 2015-06-03

**Authors:** Ted W. Simon, Robert A. Budinsky, J. Craig Rowlands

**Affiliations:** 1 Ted Simon LLC, Winston, GA, United States of America; 2 The Dow Chemical Company, Toxicology and Environmental Research & Consulting. Midland, MI, United States of America; Nihon University School of Medicine, JAPAN

## Abstract

A stochastic model of nuclear receptor-mediated transcription was developed based on activation of the aryl hydrocarbon receptor (AHR) by 2,3,7,8-tetrachlorodibenzodioxin (TCDD) and subsequent binding the activated AHR to xenobiotic response elements (XREs) on DNA. The model was based on effects observed in cells lines commonly used as *in vitro* experimental systems. Following ligand binding, the AHR moves into the cell nucleus and forms a heterodimer with the aryl hydrocarbon nuclear translocator (ARNT). In the model, a requirement for binding to DNA is that a generic coregulatory protein is subsequently bound to the AHR-ARNT dimer. Varying the amount of coregulator available within the nucleus altered both the potency and efficacy of TCDD for inducing for transcription of CYP1A1 mRNA, a commonly used marker for activation of the AHR. Lowering the amount of available cofactor slightly increased the EC50 for the transcriptional response without changing the efficacy or maximal response. Further reduction in the amount of cofactor reduced the efficacy and produced non-monotonic dose-response curves (NMDRCs) at higher ligand concentrations. The shapes of these NMDRCs were reminiscent of the phenomenon of squelching. Resource limitations for transcriptional machinery are becoming apparent in eukaryotic cells. Within single cells, nuclear receptor-mediated gene expression appears to be a stochastic process; however, intercellular communication and other aspects of tissue coordination may represent a compensatory process to maintain an organism’s ability to respond on a phenotypic level to various stimuli within an inconstant environment.

## Introduction

As science begins to comprehend the workings of transcription through new techniques such as microarrays, chromatin immuno-precipitation (ChIP), and fluorescence visualization techniques, the overall and inescapable conclusion is that the inside of the nucleus is a very busy place. Transcription factors appear to interact with DNA via a number of interdependent mechanisms that occur on multiple time scales, and these complex dynamics may be important for producing appropriate and coordinated gene expression programs;[1] these mechanisms include chromatin and nucleosome remodeling, modulation of RNA polymerase activity, and alterations in epigenetic features including acetylation and methylation. ChIP methods provide a population-level approach to the interaction of proteins with DNA and show fluctuations of DNA binding on a time scale of minutes or hours. Single cell methods typically use fluorescence microscopy and, in contrast with the ChIP results, suggest that interactions of regulatory proteins with DNA are short-lived with dwell times on the order of milliseconds.[2–6] Multiple and differing time scales and the number of unknown features of transcriptional regulation present a challenge to the development of gene expression models.

The basic helix-loop-helix Per-ARNT-Sim (bHLH-PAS) family of transcription factors occurs ubiquitously in eukaryotes. [7–9]. Cells rely on these transcription factors that dimerize and then bind to response elements on DNA. The AHR is a member of the PAS Superfamily of proteins that plays a role in the detection of and adaptation to environmental change. The name “PAS” derives from the three founding members of the family, PER, ARNT and SIM.[7] Unlike a number of the nuclear receptor transcription factors with identified endogenous ligands, e.g, hormones like corticosteroids, estrogen, testosterone, the AHR PAS protein is a transcription factor without known endogenous or natural ligands or a well-defined physiological role. [8–10] Also, the AHR has distinct evolutionary origins and protein folding patterns. Up to now, the transcriptional response to AHR activation has been investigated largely by focusing on toxicity associated with chlorinated polyhalogenated hydrocarbons and not the modulatory role of natural or endogenous ligands in various tissues for either greater understanding of normal physiology or pharmaceutical development.[11–13]

The molecular machinery of transcription also includes a number of cofactors.[14, 15] The various functions of these cofactors include chromatin remodeling, histone modification, scaffolding to enable binding of yet other cofactors, and other necessary functions[16]. For example, the Mediator cofactor is a large multi-subunit complex that produced transcription in yeast and mammalian cell extracts reconstituted with RNA polymerase and other initiation factors. Mediator appears to facilitate chromatin looping. [17, 18] Recently, the idea of “transcription factories” has been advanced; these transcription factories are assemblies of nuclear receptors, cofactors, RNA polymerases, and other co-regulatory proteins.[19–23] This organization would maximize the shared utility of transcriptional resources.

Despite these mechanisms for resource allocation, competition for transcriptional resources among independent pathways of gene expression has been observed for a number of years.[24–28] In the case of ligand-activated nuclear receptors such as the aryl hydrocarbon receptor (AHR), a plethora of molecular interactions occur in simply moving the ligand-bound receptor to the nucleus where it can dimerize with the aryl hydrocarbon nuclear translocator (ARNT) and then the liganded dimer functions as a transcription factor.[29–33]

The aryl hydrocarbon receptor (AHR) is one of the most intensively studied transcription factors. [13] After binding to a ligand, the AHR moves into the nucleus. Once there, the liganded AHR sheds the chaperone proteins including a heat shock protein (HSP90), the phosphoprotein p23, and the hepatitis-B X-associated protein 2 (XAP2).[34] In the nucleus, after losing the chaperones, the AHR binds to its heterodimer partner, ARNT.[35] The liganded AHR-ARNT heterodimer recruits a number of transcription cofactors; these include steroid receptor coactivator 1 (SRC1), the nuclear receptor coactivator protein (NCoA), the histone acetyl transferase p300/CBP, and the thyroid hormone-associated protein (TRAP220) subunit of the Mediator complex.[36–38]

Recently, there has been much discussion about nonmonotonic dose-response curves (NMDRCs), especially with regard to endocrine disrupting chemicals (EDCs).[39–45] A highly precautionary non-threshold framework approach to the regulation of presumed EDCs by the European Union prompted a letter criticizing this approach from editors of 18 prominent toxicology journals.[46] Evolutionarily, the ability to distinguish biologically relevant signals produced by endogenous hormones from signals produced by exogenous chemicals that may have weak hormonal effects—or no effect at all—would be an adaptive trait. The ability to distinguish endogenous from exogenous signals is inherent in the differences in affinity of a receptor for various ligands and the principles of chemical mass action.[47] Modeling chemical kinetics with a macroscopic approach that uses time-dynamic differential equations cannot address the stochastic nature of gene expression that has motivated concern about the lack of knowledge of potential gene expression effects in the low dose region [36, 37].

Here, we chose to examine the binding of a ligand to a nuclear receptor and subsequent transcription of mRNA to determine the likelihood of low dose effects. To do so, a model of AHR-mediated transcription of mRNA from cytochrome p450 1A1 (*CYP1A1*) gene was developed. The model uses the Gillespie stochastic simulation algorithm (SSA) [48, 49] to keep track of the changes in the number of molecules of the various reactants and products within a single cell. The stochastic nature of the model is an attempt to capture the transcriptional “noise” that may underlie some NMDRCs and provides another line of evidence to determine whether the concerns about NMDRCs and potential low-dose effects are indeed warranted.[41, 46, 50–53]

While this model can faithfully reproduce some actual measurements of AHR activation and resulting transcriptional activity, the real benefit of this exercise is the increase in understanding of the process of ligand-activated transcription. While this model may not be generalizable to all nuclear receptors and co-regulatory proteins, competition for cellular transcription resources demonstrated by the model results has been observed for other nuclear receptors. In fact, competition for cellular transcription resources is one proposed mechanism that may account for thresholds and NMDRCs in ligand-induced transcriptional responses. Regarding this competition, the relative scarcity of transcription cofactors, RNA polymerase and other transcription-associated molecules within the nucleus or “transcription factory” is increasingly recognized as a mechanism for altering gene expression.[24–28, 54–56] Competition may be the reason that these scarce resources are organized into “transcription factories” to be shared amongst a number of genes.[19, 20, 57, 58]

The model is used to demonstrate that the efficacy and potency of transcriptional responses may vary based on the availability of transcriptional resources. In some circumstances, the modeled transcriptional response shows a biphasic response with decreasing transcription at higher doses. This phenomenon, known as “squelching” has been observed for a number of transcriptional responses.[59–63] Squelching is repression of transcription at high concentrations of ligand by sequestering limiting components (e.g. coactivators) required for transcriptional activation away from the promoter in the affected gene.[63]

## Methods

The model simulates the effect of 2,3,7,8-tetrachlorodibenzodioxin (TCDD) on transcription in T47D cells.[64] The model includes binding of ligand to AHR, its movement into the nucleus and binding to the aryl hydrocarbon nuclear translocator (ARNT), binding of a generic cofactor to the heterodimer, binding of the activated heterodimer to xenobiotic response elements (XREs) associated with the *CYP1A1* gene and those associated with other AHR-induced genes, binding of RNA polymerase, transcription initiation and termination.

### Details of the Model

The model was developed from details of the well-known response of many cells to TCDD application. The model was based on ChIP experiments using T47D cells.[37, 64]

TCDD enters the cell by diffusion and once inside binds to the AHR. The ligand-bound receptor can either undergo degradation or move to the nucleus where it binds to ARNT to form a heterodimer. The ligand-bound AHR can also undergo degradation in the cytoplasm. AHR is newly synthesized at a constant rate.

Ligand-bound AHR can also move to the nucleus where it can bind to ARNT or undergo degradation. The ligand-bound dimer bind a generic cofactor to form a transcription complex. The complex can then bind to xenobiotic response elements on DNA (XREs). Two of these XREs are the TATA box and enhancer region associated with *CYP1A1*. [37] These response elements are referred to as “XREs” throughout this paper as many different xenobiotic chemicals in addition to dioxins bind to the AHR. [65, 66]

The transcription complex, consisting of AHR, ARNT and the cofactor, is also capable of binding to other AREs regulating other genes. The model assumed 470 AREs for other genes from estimates ranging between 400 and 900.[67–70]

The exact number of available RNA polymerase molecules available for AHR-activated gene expression is not known. Some RNA polymerase molecules will be bound to chromatin and transcribing genes associated with normal maintenance of the cell. Kimura et al. (1999) used saponin lysis to release soluble RNA polymerase molecules from HeLa cells and observed between 2000 and 4000 soluble molecules of RNA Pol II that were the size expected of the holoenzyme.[71] Hence, a value of 4000 molecules of RNA polymerase was assumed to be the upper limit for that available for AHR-mediated transcription.

Once the transcription complex is bound to an XRE, RNA polymerase II can also bind. Then the XRE-bound transcription complex can undergo transcription initiation. Once initiated, the complex is capable of transcription. ARNT may dissociate and transcription may still continue.[37] Transcription is terminated in a separate reaction. Within the transcription complex, another co-regulator protein could reversibly bind to the initiated transcription complex and slightly increase the rate of transcription.

We considered inclusion of negative feedback by the aryl hydrocarbon receptor repressor (AHRR) in the model.[72] However, the role of the AHRR is not sufficiently well understood that details of its action could be reproduced.[73] Further, AHRR may not play a role in the modeled response: in MCF-7 cells, similar in origin to T47D cells modeled here, AHRR protein expression in response to TCDD is not measurable before 24 hours and the simulation time here was six hours.[64, 73]Also, AHRR expression does not correlate well with TCDD-induced expression of CYP1A1 mRNA in a variety of mouse tissues and in human dermal fibroblasts.[74, 75]

Transcription itself was modeled with irreversible mass action kinetics. Any transcribed mRNA was subject to either degradation or export from the transcription complex. The kinetics of transcription are complex and include both early termination, pauses and varying rates of nucleotide addition to the end of the message.[76–84] The model includes four states of the transcription complex capable of producing mRNA, each state having a slightly different rate of transcription. These four states are included in the model in an attempt to reproduce, at some level, the complexity of transcription.

The model consists of 4 compartments, 29 molecular species, and 32 reactions. Full details of the model are provided in the Supporting Information.

### Simulation Methods

The model was developed and exercised with the Simbiology module of MATLAB. Simbiology automates the use of compiled executable files and the model runs were much faster than without compilation. Model code is available from the corresponding author and in the SI.

The simulation method used here is direct method (DM) of Gillespie’s stochastic simulation algorithm (SSA).[49] This method relies on the fact that for any biochemical reaction (e.g., ligand binding), the time to the next transition will be an exponentially distributed random variable with a mean value equal to the reciprocal of the rate constant. The Gillespie SSA is fully implemented in Simbiology.[85] As noted, the use of ordinary differential equations (ODEs) does not capture the stochastic nature of responses. Knowledge of the complexity of events occurring in the crowded space of the nucleus remains incomplete.[1] However, the use of propensities as stochastic rate constants likely comes closer to reality than macroscopic kinetics.

For simple ligand binding, two mass action reactions are involved can be shown as reversible as follows:

$$\left[AHR\right]+\left[TCDD\right]↔\left[AHR∙TCDD\right]$$

The mass action expression for the forward reaction and the forward rate constant is in units of (conc.-time)^-1^. Because there is only a single species participating in the backward reaction the rate constant is in units of time^-1^. To use Gillespie’s method, the macroscopic rate constants must be converted to propensities. The propensity of a reaction is defined as the probability of the reaction occurring in the next time interval. [48, 49, 86] The time interval to the next transition is an exponentially distributed random variable; the mean of this distribution is the reciprocal of the product of the rate constant and the concentration of one or more reactants. [48]

100 runs of the model were conducted for each TCDD concentration. The simulation time was 6 hours and time unit used was one second. Because the time vectors associated with each run were different because of the probabilistic calculation for the propensity, linear interpolation was used to obtain the mean and variance of the numbers of molecules for each species throughout the course of the run from the 100 runs that represent a population of 100 cells.

## Results

### Comparison of Measured vs. Modeled Transcription

**Fig 1:**
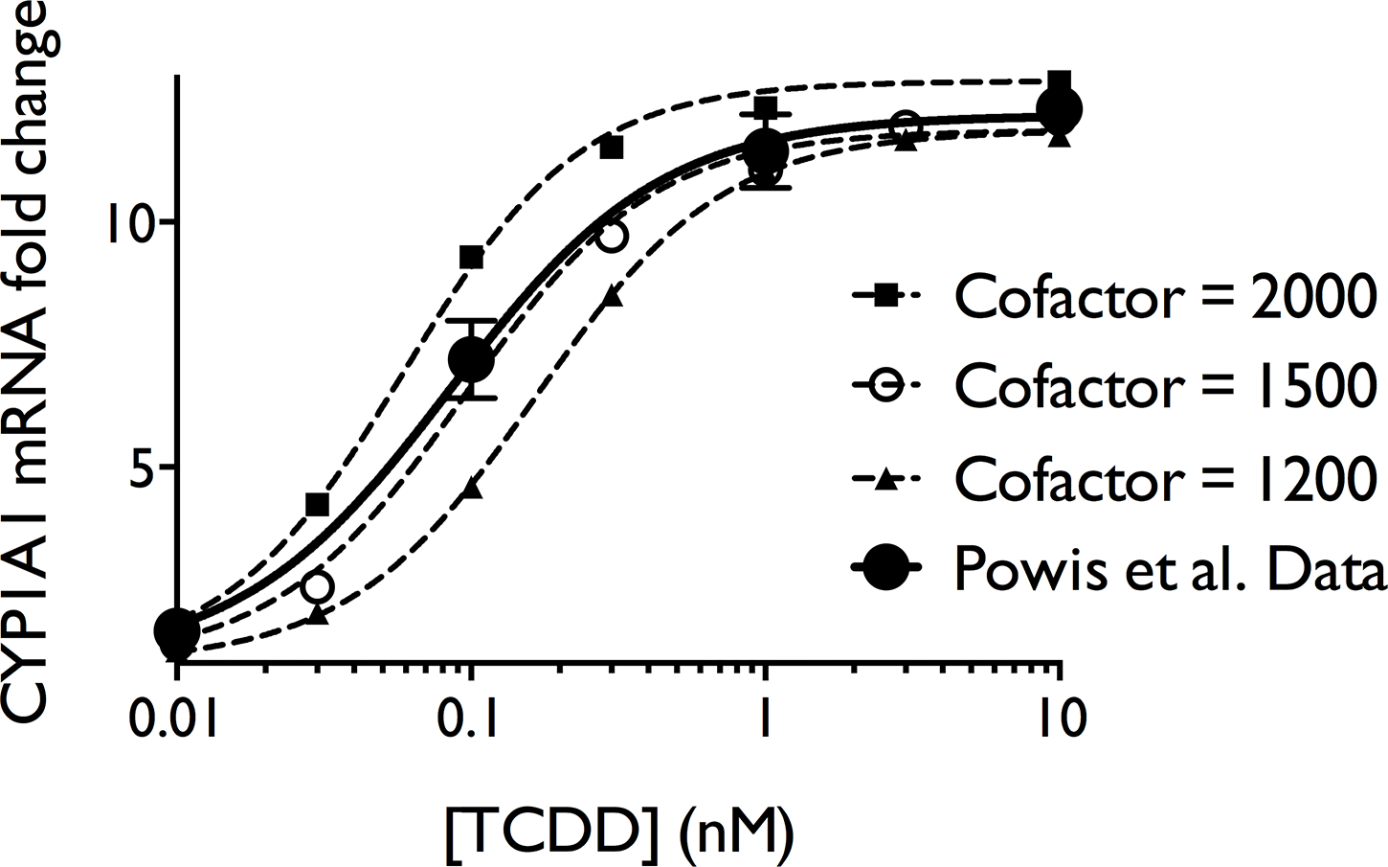
Measured and modeled transcriptional dose-response to TCDD. The larger filled black circles show the transcriptional response of CYP1A1 at 6 hours in T47-D cells. These data were digitally extracted from Fig 1 in Powis et al. (2011). [64] The smaller symbols and dotted lines show the modeled transcriptional dose response at three different amounts of cofactor present. When 1500 molecules of cofactor were present, the modeled response is very similar to the observed response in T47-D cells by Powis et al. (2011). [64]

The model was exercised with a range of TCDD concentrations and a range of cofactor amounts. The model faithfully reproduces the transcriptional dose-response for CYP1A1 mRNA at 6 hours from Powis et al. (2011)[64] when 1500 molecules of cofactor, 1535 molecules of competing non-AHR binding proteins (Other) and 4000 molecules of RNA polymerase are present in the nucleus (Fig 1). Also shown are the modeled transcription dose-response curves for 2000, 1500 and 1200 molecules of cofactor. Fitting the Powis et al. (2011) data to a Hill dose-response model yielded an EC50 value of 0.086 nM and a Hill coefficient of 1.12. Fitting the model results with 1500 molecules of cofactor yielded an EC50 value of 0.094 nM and a Hill coefficient of 1.35 (Table 1) and the two plots are almost indistinguishable (Fig 1). Estimates of transitional dose values[87, 88] were determined by baseline projection from the ligand concentration needed to produce 21% of the maximal response (EC21) using S4 Equation in S1 File (Table 1).

**Table 1:** Hill equation fits of modeled data at a range of cofactor amounts along with the fit to the transcriptional response in Fig 1 in Powis et al. (2011) [61].

Altered Amount (molecules)	Bmax (fold change) (mean ± SE)^1^	Hill Coefficient (mean ± SE)^1^	EC50 (nM) (mean ± SE)^1^	Transitional Dose Value from EC21^2^ (nM)
**Powis et al. data**	11.18 ± 0.1880	1.212 ± 0.1202	0.08508 ± 0.00592	0.02580
**Modeled results from altering cofactor amounts (Fig 1 and Fig 3A)**				
**200**	1.421 ± 0.0566	1.826 ± 0.2768	0.09873 ± 0.0091	0.04275
**400**	2.547 ± 0.1550	1.637 ± 0.3258	0.1050 ± 0.0153	0.04139
**800**	10.92 ± 0.2650	1.252 ± 0.0431	0.3134 ± 0.0164	0.07515
**1000**	10.33 ± 0.1531	1.418 ± 0.0684	0.2101 ± 0.0088	0.06443
**1200**	10.87 ± 0.0583	1.360 ± 0.0312	0.1659 ± 0.00327	0.05148
**1300**	10.94 ± 0.1599	1.310 ± 0.0826	0.1319 ± 0.0074	0.04105
1500	**10.87** ± **0.2173**	**1.350** ± **0.1243**	**0.09446** ± **0.00756**	**0.03166**
**2000**	11.87 ± 0.1820	1.431 ± 0.1089	0.05903 ± 0.00384	0.02182
**3000**	11.37 ± 0.2045	1.437 ± 0.1316	0.04963 ± 0.00386	0.01862
**6000**	11.62 ± 0.2525	1.519 ± 0.1722	0.05223 ± 0.00481	0.02054
**Modeled results from altering the number of Other cofactor binding sites (Fig 3B)**				
**50000**	9.335 ± 0.3997	1.784 ± 0.2106	0.1746 ± 0.0156	0.06815
**20000**	8.889 ± 0.2663	1.757 ± 0.1422	0.1782 ± 0.01112	0.07346
**10000**	10.07 ± 0.3268	1.565 ± 0.1191	0.1978 ± 0.0135	0.06736
**7500**	10.78 ± 0.3106	1.358 ± 0.07192	0.2395 ± 0.0149	0.06803
**2500**	10.60 ± 0.03626	1.276 ± 0.01765	0.1702 ± 0.00217	0.04929
**1535**	**10.87** ± **0.2173**	**1.350** ± **0.1243**	**0.09446** ± **0.00756**	**0.03166**
**750**	11.60 ± 0.1565	1.351 ± 0.08818	0.05439 ± 0.003197	0.01913
**250**	11.79 ± 0.1223	1.285 ± 0.0618	0.05867 ± 0.002662	0.01952

Fitted parameters are shown as the best-fit value ± standard error. The upper part of the table shows fits for a series of varying cofactor amounts. The lower part of the table shows fits for a series of varying competing non-AHR cofactor binding sites (Other). Fitting was conducted with Graphpad Prism. The rising portion of the curve was fit. It was not possible to obtain a Hill equation fit to the modeled results at 60 molecules of cofactor. The lower part of the table shows the effect of changing the number of competing binding sites for the cofactor.

^1^ For 1000 cofactor molecules, points below 3 nM TCDD were fit, and for 800 molecules and lesser amounts, points below 1 nM TCDD were fit. It was not possible to obtain a Hill equation fit to the modeled results at 60 molecules of cofactor.

^2^ Transitional dose values (TDVs) as a measure of threshold were estimated by projecting to the background response using the methods for the Hill model described in Simon et al., (2014). [78] The equations for estimating TDVs using background projection from Simon et al., 2014 are shown in Equation D in S1 File.

### Comparison of Measured and Modeled Binding to DNA

**Fig 2:**
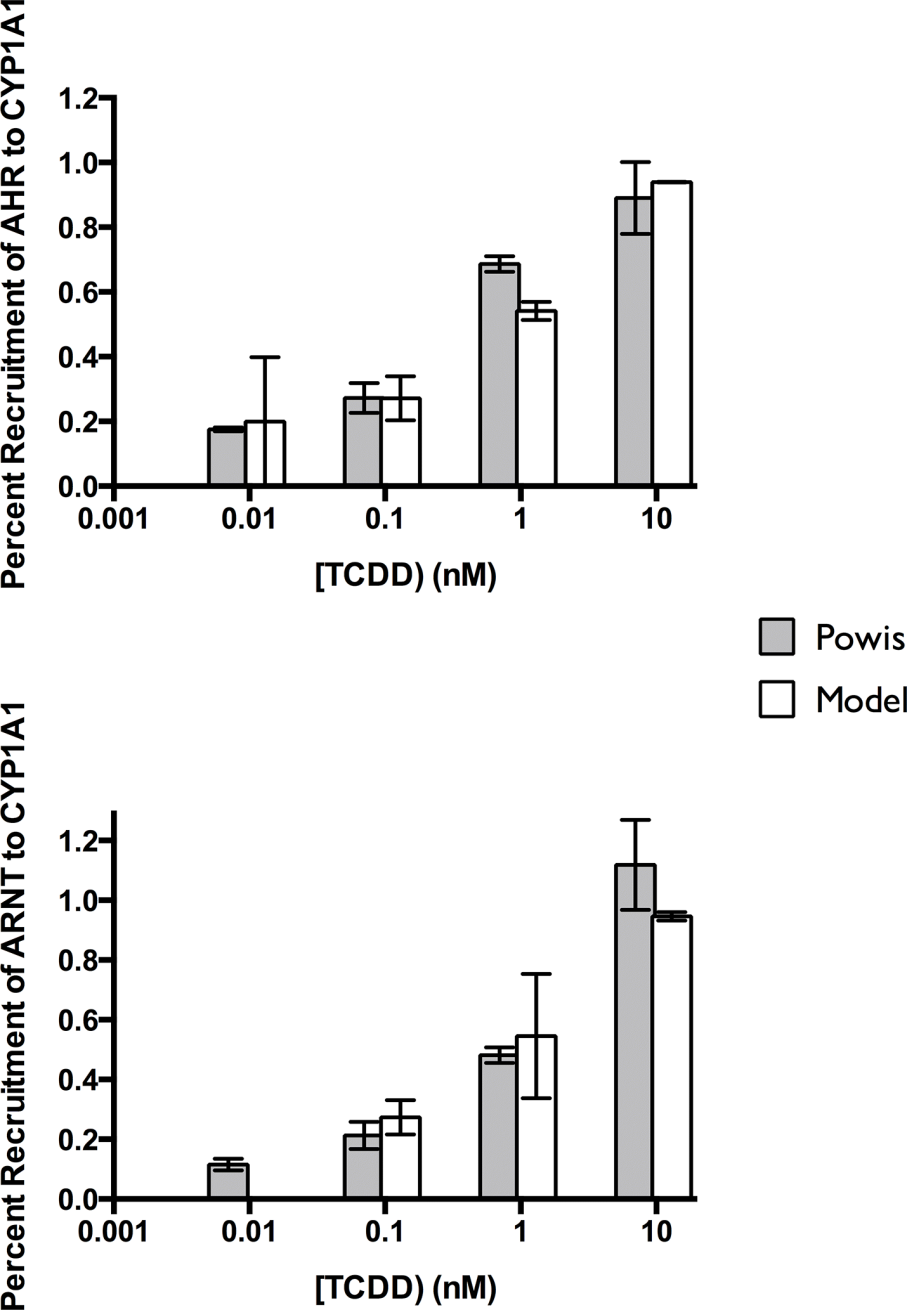
Comparison of ChIP results for AHR and ARNT from Fig 2A of Powis et al. (2011) [64] with those of the model. ChIP results were estimated with Equation A and Equation B in S1 File. **(A)** Percent recruitment of AHR to CYP1A1; **(B)** Percent recruitment of ARNT to CYP1A1.

A comparison of measurements of binding of both AHR and ARNT to XREs associated with *CYP1A1* measured by ChIP experiments and corresponding model results is shown in Fig 2. The modeled result was very close to the measured ChIP results.

The value of percent recruitment represents the fraction of a particular transcription factor bound to DNA.[64] The calculation of percent recruitment from the model results is shown in the Equation A in S1 File.

### The Effect of Altering Cofactor Concentrations or the Number of Binding Partners for the Cofactor other than AHR-ARNT

**Fig 3:**
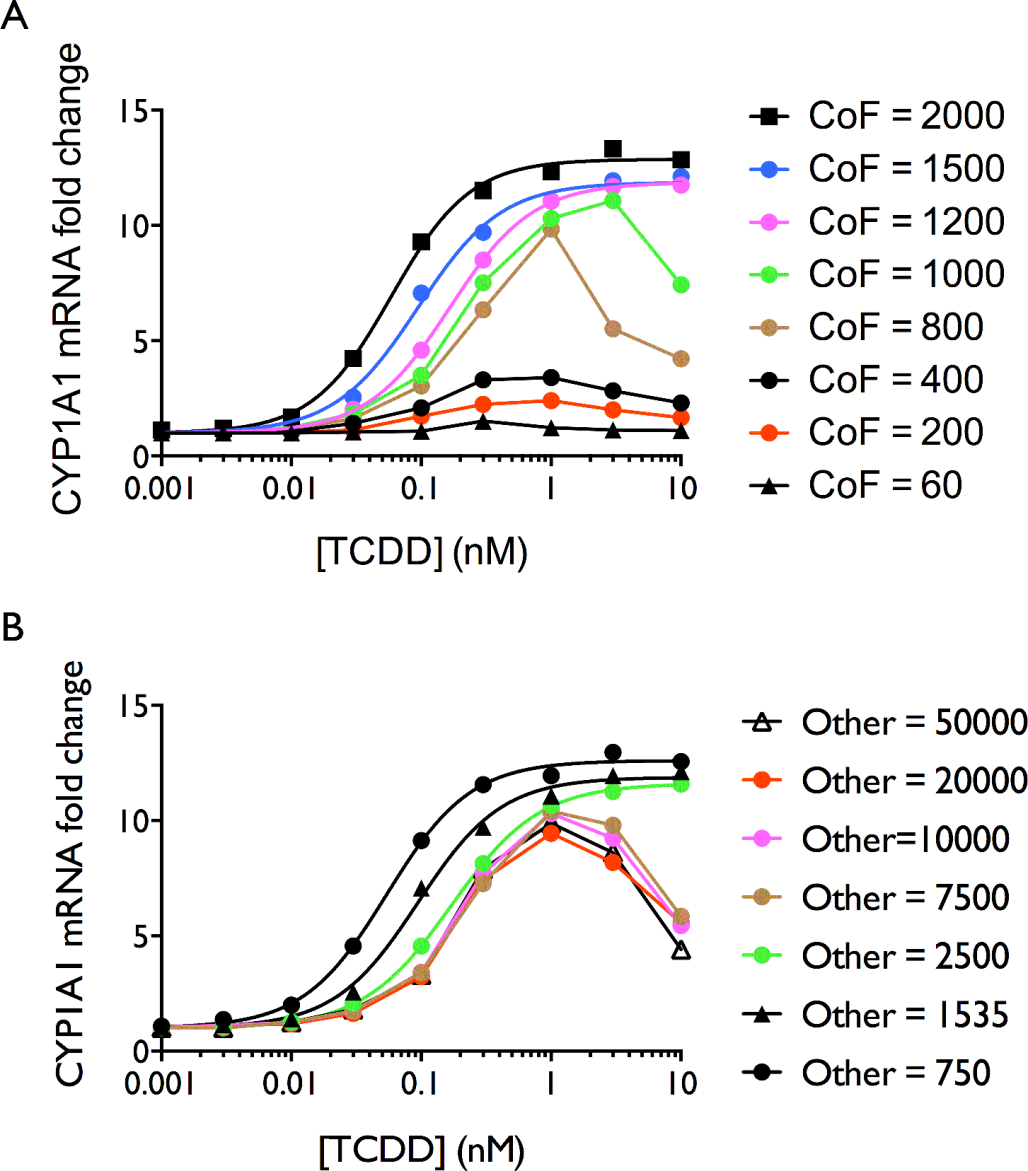
Modeled transcriptional dose-response plots at varying amounts of cofactor and non-AHR-ARNT cofactor binding sites. (**A**) Reduction in the amount of cofactor (CoF) at a constant concentration/amount of competing non-AHR binding proteins (1535 molecules). The modeled response and Hill equation fits are shown for cofactor amounts of 2000, 1500 and 1200 molecules. At 1000 molecules of cofactor and less, squelching was apparent, shown by a reduction in the responses at higher TCDD concentrations and the biphasic appearance of the DR curves. (**B**) Increasing the amount of competing non-AHR binding proteins (Other) also produced a squelching-like response at high ligand concentrations with squelching occurring at 7500 or more molecules of non-AHR cofactor binding proteins. The amount of cofactor was kept constant at 1500 molecules. The Hill equation fits are shown for competing non-AHR binding site (Other) amounts of 2500 or less.

Squelching is apparent at cofactor amounts of 1000 molecules or less and appears as a reduction in the response at TCDD concentrations of 1 nM or greater (Fig 3A). The EC50 can change approximately four fold between cofactor amounts of 1000 and 3000 molecules without a change in the efficacy of the response (Table 1). When squelching occurs, the trend of EC50 values reverses and a reduction in EC50 values, i.e. higher potency, occurs with decreasing cofactor amounts when cofactor amounts are less than 1000 molecules.

Squelching is a reduction in the response at higher concentrations and results in the appearance of NMDRCs. Squelching was also observed to occur in the model in response to increasing the cofactor binding species other than AHR-ARNT (Table 1; Fig 3B) (Species #8 in Table A in S1 File). Squelching is a well-documented observation for the estrogen receptor and has been observed in many ToxCast results.[89]

Increasing the number of competing non-AHR cofactor binding sites (Other), induced squelching and NMDRCs at amounts of competing non-AHR binding sites at 7500 or more (Fig 3B).

### The Roles of Cofactor and Competition in Squelching

**Fig 4:**
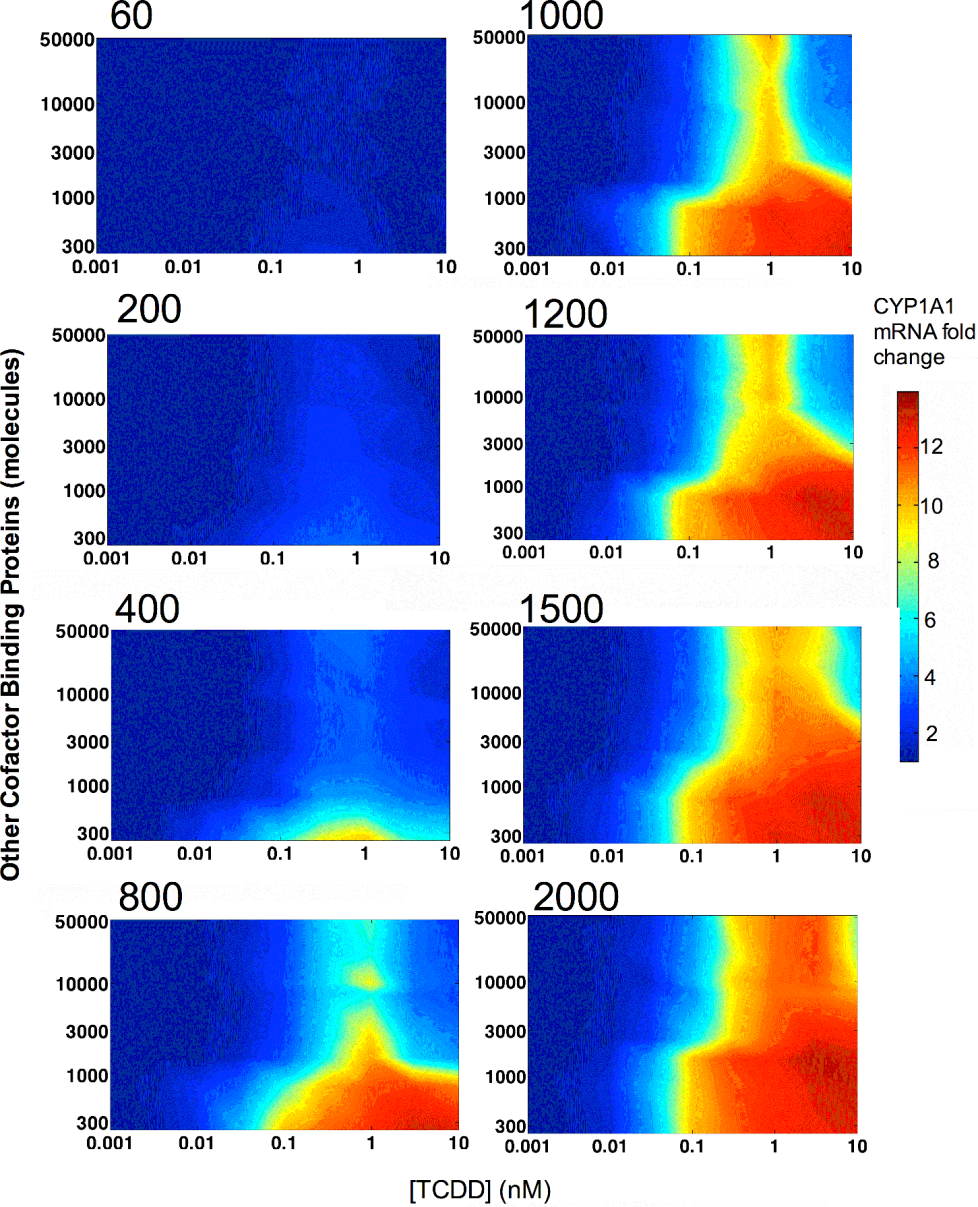
Contour plots of the modeled transcriptional responses showing the relationship between number of cofactor molecules and the number of competing non-AHR (Other) binding proteins. The x-axes show the applied concentration of TCDD. The y-axes show the amount of other binding proteins available in the cell. The fold change in CYP1A1 mRNA is represented by the colors on the plots and the color bar to the right. The number at the upper left of each plot shows the number of molecules of cofactor.

To demonstrate the interaction of cofactor availability, the model was exercised at a range of TCDD concentrations and various combinations of the amounts of cofactor and non-AHR cofactor binding proteins that would compete with the AHR-ARNT complex for available cofactor. With these model results, contour maps of the transcriptional response were developed for varying amounts of both cofactor and non-AHR cofactor binding proteins (Fig 4) (Species #7 and #8 in Table A in S1 File). Squelching is apparent at all concentrations of cofactor. Hence, squelching occurs as an interaction between the amount of cofactor and the amount of competing non-AHR binding proteins.

### The Effect of Altering Ligand Activated AHR on Dose-Response Curves and Squelching

**Fig 5:**
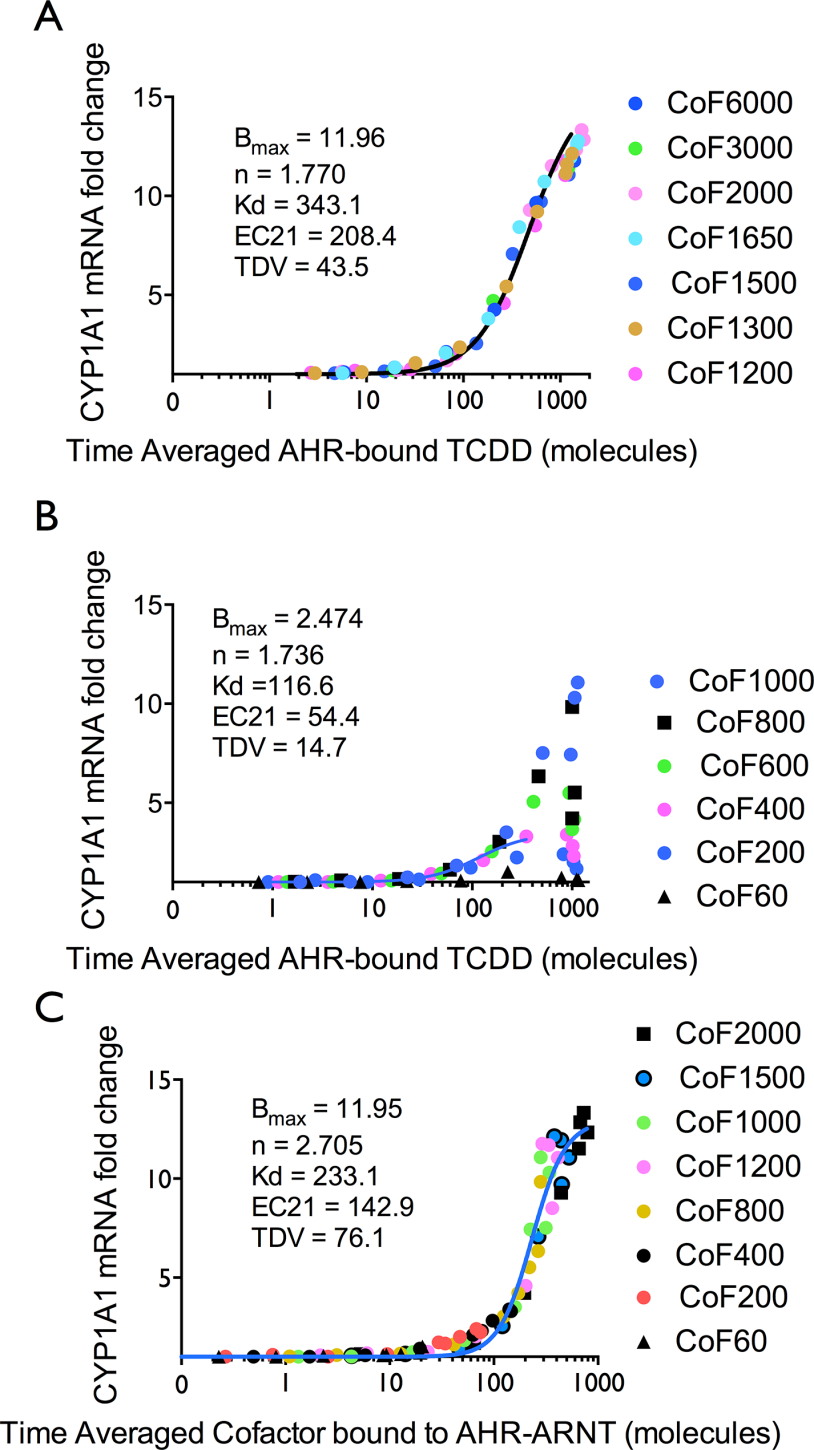
Transcriptional dose-response using time-averaged species from the model results to demonstrate that squelching occurs at the cofactor-binding step. Each plot was fit to a Hill function (details in text) and the EC21 and transitional dose values are shown. [87, 88] **(A)** Plot of CYP1A1 mRNA fold change vs. time-averaged ligand-bound AHR for responses without squelching. **(B)** Plot of time-averaged ligand-bound AHR for responses with squelching. **(C)** Plot of time-averaged cofactor bound to AHR-ARNT and thus contributing to CYP1A1 transcription.

To explore how cofactor availability acts to produce shifts in the dose-response curves and squelching, the transcriptional response of the model was plotted against the time-averaged number of ligand-activated AHR molecules. Hence, the total number of ligand-activated AHR molecules as a time average was used as the dose term and the mRNA fold change was used as the response term. A first order Hill model was fit to the dose-response of the aggregated data for responses when no squelching was apparent (Fig 5A) and for responses showing squelching (Fig 5B). When no squelching occurred, the aggregate response was well fit by a Hill equation and the transcriptional responses were consistent at all doses of TCDD (Fig 5A). When squelching did occur (1000 cofactor molecules and less), considerable divergence in the responses to higher TCDD concentrations was observed (Fig 5B). A Hill model was fit to the data with 400 ligand-bound AHR molecules or less and the fit indicated an approximately fivefold reduction in efficacy in the squelched responses.

When the time-averaged amount of cofactor bound to AHR-ARNT anywhere in the cell (Equation C in S1 File) is used as the dose term, the responses become very different, and the aggregate dose-response, both for those squelching and non-squelching responses remain consistent and were fit to a Hill function (Fig 5C). Thus, these plots demonstrate that the locus at which squelching occurs is cofactor binding to AHR-ARNT.

## Discussion and Conclusions

### Modulation and Variability of the Dose-Response for Receptor-mediated Effects

When one observes the dose-response for the formation of all ligand-bound AHR averaged over the duration of simulation (Fig 5A; Equation B in S1 File), no squelching is observed, and the dose-response curves have sufficiently similar values for EC50 and the Hill coefficient that these parameters can be fit in common. (Fig A in S1 File). Hence, squelching occurs at a later step than ligand-receptor binding.

Lately there has been much discussion about the lack of knowledge of events in the low dose region—especially regarding binding of nuclear receptors and subsequent gene expression.[39] This model demonstrates that because of the limited availability of transcription cofactors and the stochastic nature of molecular events involved in gene expression, transitional dose values that represent response thresholds need to be considered as variable or stochastic values rather than a fixed value.[87, 88] Stochastic variation in a population of T-cells has been observed and limited numbers of regulator molecules associated with transcription has been observed to introduce noise into cellular processes.[90–92]

Histograms of transcriptional responses of all 100 cells indicate that only a single cell shows an increase in mRNA copy number at the lowest dose modeled (Fig B in S1 File). Some concern has been expressed about endocrine effects in the low dose region.[39, 41] In the low dose region, greater variability in mRNA copy number can be observed in the model results than predicted by Poisson statistics (Table E in S1 File, 4^th^ and 5^th^ columns). However, when considered the overall response of the modeled tissue or cell population, consisting of 100 modeled cells, the total number of mRNA copies is well within the expected range (Table E in S1 File, last two columns). Hence, the model results indicate that on a tissue or cell population basis, gene expression responses will likely be very small or non-existent in the low dose region and that these concerns may be misplaced.

As well as TCDD concentration, the transcriptional response depends to a fairly large extent on the availability of cofactor and other cellular resources. However, at the lowest TCDD concentration at which the model was executed, the overall response of the tissue is absent (Fig 4). The availability of cellular resources can affect both the efficacy and potency of the response and this fact should be considered for interpretation of *in vitro* high throughput assays.[89, 93, 94] Recently, assays have been developed for transcription cofactors and these may have the potential for judging the performance of *in vitro* assays that are increasingly being used for regulatory purposes.[95–97]

Because of the changing needs of organisms, the context for appropriate gene expression is inconstant, and this context, reflected by the panoply of genes expressed given the limitations on resources, will result in ever changing levels of available cofactor and thus ever changing “thresholds.” Stochastic variation in these “thresholds” combined with the difficulty in knowing what level of response is biological significant in what particular context is one of the factors that accounts for the focus on discovering meaningful methods for filtering gene expression data. [98–101]

A method for estimating transitional dose values (TDVs) for dose-response data that follows a Hill equation as potential estimates of thresholds has been developed and was used here to estimate transitional dose values for TCDD concentration thresholds for the transcriptional response (Table 1).[87, 88] In addition, TDVs were estimated for the time-averaged number of ligand-bound AHR molecules and the number of AHR-ARNT complexes with bound cofactor. The method and equations are provided in Equation D in S1 File. The calculated TDVs for TCDD concentration are shown in Table 1.

### Identity of the Cofactor and Other Binding Sites

This model was developed to explore the role of competition in ligand-activated gene transcription. Hankinson (2005) provides a list of possible candidates for the cofactor including histone acetylases, histone methyl transferases or receptor-interacting protein 140 (RIP140).[36] Recently, four cellular factors were identified in Hepa1c1c7 cells as crucial for CYP1A1 mRNA induction independent of effects on AHR expression. These factors are SIN3 homolog A (SIN3A), phosphoducin (PDC), transmembrane protein 5 (TMEM5), and the CD9 cell surface glycoprotein (CD9). [102] Although the model could be altered to include cofactor involvement in AHR induction, some cofactors such as transmembrane factor 20 (TCF20) and crystalline gamma D (CRYGD) are necessary for induction of both CYP1A1 and AHR mRNA and protein. [102]

Nuclear factor erythroid 2-realted factor 2 (Nrf2) interacts with the AHR in its role in the expression of both CYP1A1 and NQO1.[103–105] Nrf2 is a transcription factor involved in responses to oxidative and electrophilic stress, and inflammation.[106] Nrf2 interacts with both the AHR and the estrogen receptor. [107] In Nrf2-null mice, the transcriptional response to TCDD for CYP1A1 was reduced compared to that in wild-type mice. [103, 105, 107] Hence, Nrf2 is another potential candidate for the identity of the cofactor in the model.

Yet another candidate for the identity of the cofactor in the model is nuclear factor-κB (NF-κB). Inhibition of NF-κB reduced the expression of mRNA for both AHR and CYP1A1 in U-937 derived dendritic cells.[108]

2,3,7,8-Tetrachlorodibenzo-p-dioxin poly(ADP-ribose) polymerase (TiPARP, ARTD14) co-localizes with AHR in the nucleus. TiPARP enhances the production of CYP1A1 mRNA but also increases the proteolytic destruction of ligand-bound AHR. [109] This negative feedback mechanism could also be included in the model.

### Oscillations in ChIP Data

At present, data on binding of transcription factors to DNA is obtained by two primary methods—chromatin immuno-precipitation and fluorescence techniques—with the former conducted on populations of cells and the latter on single cells. These two methods give very different kinetic results. Hager (2009) has proposed that several explanations for the oscillations of transcription factor binding observed in ChIP experiments.[4] ChIP experiments are often expressed as fractional or percent recruitment to a single XRE or limited set of XREs with total chromatin input as the denominator.

Oscillations in the binding of either AHR, ARNT, cofactor or RNA polymerase to *CYP1A1* were not observed in any of the model runs.

### Modeling Transcription

Current knowledge of the process of transcription suggests it is complex and not yet well understood[78, 82–84, 110, 111] In the model, transcription of *CYP1A1* was represented by four different promoter states, each with a slightly different rate constant. This simplified model was an attempt to introduce variable transcription rates into the model. For the sake of parsimony, more complex models of transcription were not used.[83, 110, 111] Rates of mRNA export from the transcription complex and degradation within the complex were determined empirically to match mRNA fold change data at 6 hours in T47-D cells.[64] Distributions of CYP1A1 mRNA copy number per cell at various TCDD concentrations were consistent with Poisson distributions similar to that measured in other eukaryotic cells and observed by other modelers (Fig B in S1 File; Table E in S1 File).[112–115]

### Examples of Possible Competition for Cofactors

Whether ARNT is considered a transcription factor or a cofactor is somewhat moot. The basic helix-loop-helix-PAS proteins appear to have been evolutionarily conserved and serve multiple roles. ARNT dimerizes with the bHLH-PAS protein hypoxia inducible factor 1 (HIF-1) that acts to produce cellular responses to hypoxia.[36, 116] ARNT also acts as a coactivator for both ERα- and ERβ-mediated transcription.[117, 118] AHR-mediated transcription can be modulated by recruitment of a variety of cofactors including CBP/p300, p160/SRC-1, NCoA2, p-CIP and RIP140.[16, 34, 37]

Nonmonotonic U-shaped dose response curves have been shown to occur for steroid hormone-mediated gene expression. Such shapes in the dose-response occur when there exist receptors unoccupied by endogenous hormones and recruitment of cofactors by receptors bound to xenobiotic ligands is weaker than to hormone-bound receptor.[44] For the androgen receptor, the presence of mixed ligand heterodimers that are transcriptionally inactive along with homodimers with bound hormone and xenobiotic ligand lead to J-shaped dose-response curves reminiscent of hormesis.[45] Receptor dimerization appears to be an inherently non-linear process and the normal milieu of endogenous hormones along with a xenobiotic ligand produce a variety of shapes of dose-response curves.[43] The situation modeled here, competition for one or more coregulatory proteins, only occurs at high concentrations of ligand and high receptor occupancy. At low concentrations of ligand, coregulatory proteins are sufficiently available to ligand-bound receptors for normal transcriptional/signaling responses.[119, 120]

ARNT serves as a coactivator for ERα and ERβ, similar to the generic cofactor modeled here.[117] Hence, one could imagine a situation where the physiological need for estrogen-mediated gene expression might result in a competition for ARNT such that the AHR response to endogenous or exogenous ligands might be muted.

### Other Possible Uses of the Model

Transforming growth factor-β1 (TGF-β1) appears to act as an inhibitor or repressor of AHR-mediated effects in a cell line derived from human prostate cells.[121] The cross-talk between TGF-β1 and AHR is complex, but this could be explored initially with this model as a second cofactor with repressive effects.

2,3,7,8-Tetrachlorodibenzo-p-dioxin poly(ADP-ribose) polymerase (TiPARP, ARTD14) co-localizes with AHR in the nucleus. TiPARP enhances the production of CYP1A1 mRNA but also increases the proteolytic destruction of ligand-bound AHR.[109] This negative feedback mechanism could also be included in the model.

Other processes that could be included in the model are:

- Induction of AHR by including AHR synthesis dependent on AHR activation level;
- Repression of AHR by TIPARP[73] or other possible mechanisms; and,
- Transcription of other genes in the AHR core battery at different transcription rates.

While the model is somewhat complex, the actual inter-relationships between receptors involved in gene induction are even more extensive and inter-related. Extensive crosstalk and overlap exists between the AHR, ERα and Nrf2.[107] A specific model of this crosstalk could potentially be developed and could be used to explore the effects of ligands for each of these receptors presented alone or in combination.

### Stochastic gene expression in evolution

Stochastic effects resulting from transcription factor binding likely produce heterogeneity in gene expression across cells within organs or tissues in metazoans. This heterogeneity could result from stochasticity in gene activation and inactivation processes and could be an overall compensatory mechanism for the limitations on resources within a single cell. Hence, at the organ tissue levels, the ability to respond to stimuli or environmental changes by gene expression would be maintained.[113, 122–124]

Within a single cell, a small number of mRNA molecules can potentially be amplified into a sufficient number of protein molecules to produce a phenotypic change in that cell. Cell-to-cell signaling may occur either by extracellular chemical signals such as cytokines or through gap junctions between adjacent cells. One possible function of such signaling could be to regulate gene expression on a tissue-wide basis. The fact that gene expression in metazoans occurs within nuclear structures that maintain locally high concentrations of transcription components is testament to these resource limitations.[19, 20, 125] The model results suggest that these resource limitations would limit any tissue-level phenotypic responses.

If the oscillations observed in ChIP data represent different levels of nuclear receptor binding in cell populations within tissues, this could be another aspect of this mechanism of tissue-wide coordination.[4] What these model results also suggest is that generalizations about mechanisms or even responses across different levels of biological organization (e.g., molecule, cell, tissue, organism) may lead to faulty conclusions and begs the question: What else is going that leads to an observed phenotypic change?

## Supporting Information

S1 File Supporting Information. This file contains: Code A, MATLAB Script for Model Runs. Code B, SBML Code for the Model. Equation A, Calculation of Percent Recruitment from the model results. Equation B, Calculation of Time-Averaged Numbers of ligand-bound AHR. Equation C, Calculation of Time-Averaged Numbers of cofactor bound to AHR-ARNT. Equation D, Equations used for Baseline Projection to determine Transitional Dose Values from Simon et al., (2014). Fig A, Dose-response to the number of ligand-bound AHR molecules as a function of TCDD concentration and fitted Hill functions. Fig B, Histograms of CYP1A1 mRNA copy number induced by TCDD. Table A, Compartments and Species. Table B, Reactions and Rate Constants. Table C, Comparison of classical rate constants, molecular rate constants and propensities. Table D, Hill Model fits of the Percentage of Cells with one or more CYP XRE bound. Table E, Goodness of fit of Poisson distribution for modeled CYP1A1 mRNA copy number.
(DOCX)
