# Supplementary material for: A Model for Aryl Hydrocarbon Receptor-Activated Gene Expression Shows Potency and Efficacy Changes and Predicts Squelching Due to Competition for Transcription Co-Activators
Source: PLoS One. 2015 Jun 3;10(6):e0127952. doi: 10.1371/journal.pone.0127952 (PMC4454675; doi:10.1371/journal.pone.0127952)
Supplement: S1 File — This file contains: Code A, MATLAB Script for Model Runs. Code B, SBML Code for the Model. Equation A, Calculation of Percent Recruitment from the model results. Equation B, Calculation of Time-Averaged Numbers of ligand-bound AHR. Equation C, Calculation of Time-Averaged Numbers of cofactor bound to AHR-ARNT. Equation D, Equations used for Baseline Projection to determine Transitional Dose Values from Simon et al., (2014). Fig A, Dose-response to the number of ligand-bound AHR molecules as a function of TCDD concentration and fitted Hill functions. Fig B, Histograms of CYP1A1 mRNA copy number induced by TCDD. Table A, Compartments and Species. Table B, Reactions and Rate Constants. Table C, Comparison of classical rate constants, molecular rate constants and propensities. Table D, Hill Model fits of the Percentage of Cells with one or more CYP XRE bound. Table E, Goodness of fit of Poisson distribution for modeled CYP1A1 mRNA copy number. (DOCX) [file pone.0127952.s001.docx]

# Supporting Information

**Table A. Compartments and Species**

| Compartments | | |
| --- | --- | --- |
| Diffusion Volume (L) | | 3E-10 |
| Cytoplasm (L) | | 1.25E-12 |
| Nucleus (L) | | 5.1E-13 |
| Transcription Complex (L) | | 2.0E-14 |
| Species | | |
| TCDD | Diffusion Volume | 0.001, 0.003, 0.01, 0.03, 0.1, 0.3, 1, 3, 10 nM  768, 2300, 7662, 22996, 76652, 229960, 766520, 2299600, 7665200 molecules |
| TCDD (1) | Cytoplasm | 0 (initial concentration) |
| AHR (2) | Cytoplasm | 11.06 nM, 8333 molecules |
| AHR-TCDD (3) | Cytoplasm | 0 (initial concentration |
| AHR-TCDD (4) | Nucleus | 0 (initial concentration) |
| ARNT (5) | Nucleus | 12.24 nM, 3686 molecules |
| AHR-ARNT (6) | Nucleus | 0 (initial concentration) |
| CoF (7) | Nucleus | 0.066, 0.199, 0.664, 1.99, 6.64, 19.93 nM  20, 60, 200, 400, 800, 1000, 1200, 1500, 2000 molecules |
| Other CoF binding species (8) | Nucleus | 5.1 nM or 1535 molecules |
| AA-CoF (9) | Nucleus | 0 (initial concentration) |
| Other-CoF (10) | Nucleus | 0 (initial concentration |
| CYP-DRE (11) | Nucleus | 4 molecules (Matthews et al., 2005) |
| DRE (12) | Nucleus | 470 molecules (Lo and Matthews, 2012) |
| CYP-AA-CoF (13) | Nucleus | 0 (initial concentration) |
| DRE-bound (14) | Nucleus | 0 (initial concentration) |
| RNA Polymerase (15) | Nucleus | 4000 molecules |
| CYP-AA-CoF-Pol (16) | Nucleus | 0 (initial concentration) |
| mRNA (17) | Nucleus | 1 (initial concentration) |
| DRE-AA-CoF-Pol (18) | Nucleus | 0 |
| Initiated CYP (19) | Transcr. Complex | 0 |
| Initiated CYP ARNT lost (20) | Transcr. Complex | 0 |
| mRNA_t (21) | Transcr. Complex | 0 |
| Initiated DRE (22) | Transcr. Complex | 0 |
| Initiated DRE ARNT lost (23) | Transcr. Complex | 0 |
| Coregulator (24) | Transcr. Complex | 100 molecules |
| CYP-init-CoReg (25) | Transcr. Complex | 0 |
| CYP-init-CoReg ARNT lost (26) | Transcr. Complex | 0 |
| Other CoReg site (27) | Transcr. Complex | 1000 molecules |
| CoReg bound at other site (28) | Transcr. Complex | 0 |

**Code A. MATLAB Script for Model Runs**

%%

%% Load SimBiology project.

clf;

clc;

clear;

out = sbioloadproject('/Volumes/BLUE_FLASH/MATLAB/Gene-exp-TCDD/TCDD_simp3_123013.sbproj');

m1 = out.m1;

%% Initialize configset for analysis run.

cs = getconfigset(m1, 'default');

set(cs, 'SolverType', 'ssa');

set(cs.SolverOptions, 'LogDecimation', 200);

set(cs, 'StopTime', 21600);

% RUNSCAN run a scan over parameter, species or compartment values.

% For each scan iteration, simulate the SimBiology model, m1.

% Assign values for scan.

% Set CoF amount

%

%*************************************

% Check filename for correct saving

%*************************************

%%

set(m1.Species(1), 'InitialAmount', 2299600.0); % TCDD in diffusionVol

set(m1.Species(3), 'InitialAmount', 8330.0); % AHR, default=8330

% Species(6) = ARNT = 100, 300, 1000, 3000, 10000

set(m1.Species(6), 'InitialAmount', 3686.0); % ARNT, default=3686

% Species(8) = CoFactor = 20, 60, 200, 400, 600, 800, 1000, 1200, 1500, 2000, 3000 molecules

% 1500 is the value that matches the Powis gene expression data

set(m1.Species(8), 'InitialAmount',3000.0); % CoF

% Species(9) = Other = 250, 750, 1535, 2500, 7500, 10000, 20000, 50000

set(m1.Species(9), 'InitialAmount', 2500.0); % Other, default=1535

% Species(16) = RNA_Pol = 20, 50, 200, 500, 2000, 5000

set(m1.Species(16), 'InitialAmount',4000.0); % RNA_Pol, default TBD, start with 4000

% Speciex(13) = nonCYP DREs = 470.0)

set(m1.Species(13), 'InitialAmount', 470.0); % DRE (nonCYP) default = 500

% Species(25) = CoReg1 = 2, 5, 10, 20, 50, 100, 200, 500, 1000 default = 100

set(m1.Species(25), 'InitialAmount',100.0); % CoReg1, the CoR inside the transcriptome

% Species(28) = CoR_Other = 2, 5, 10, 20, 50, 100, 200, 500, 1000, 2000, 5000, 10000 default = 1000

set(m1.Species(28), 'InitialAmount',1000.0); % CoR_Other, other sites for CoReg binding

%% set parameters

set(m1.Reactions(1).KineticLaw.Parameters(1), 'Value', 8.E-07); % kdiff going in 8E-07

set(m1.Reactions(2).KineticLaw.Parameters(1), 'Value', 5.3E-07); % kf1 bind to AHR 5.3E-07

set(m1.Reactions(2).KineticLaw.Parameters(2), 'Value', 0.002); % kb1 undbind from AHR 0.002

set(m1.Reactions(3).KineticLaw.Parameters(1), 'Value', 0.00151); % kin AHR-T to nucleus 0.00151

set(m1.Reactions(3).KineticLaw.Parameters(2), 'Value', 8.333E-05); % kout AHR-T from nucleus 8.333E-05

set(m1.Reactions(12).KineticLaw.Parameters(1), 'Value', 0.4); % k_Polbind

set(m1.Reactions(12).KineticLaw.Parameters(2), 'Value', 0.145); % k_Polunbind

set(m1.Reactions(13).KineticLaw.Parameters(1), 'Value', 0.04); %k_DPolbind

set(m1.Reactions(13).KineticLaw.Parameters(2), 'Value', 0.145); % k_DPolunbind

set(m1.Reactions(14).KineticLaw.Parameters(1), 'Value', 0.03); % k_init

set(m1.Reactions(16).KineticLaw.Parameters(1), 'Value', 0.019); % k_term

set(m1.Reactions(17).KineticLaw.Parameters(1), 'Value', 0.01); % k_Amterm

set(m1.Reactions(28).KineticLaw.Parameters(1), 'Value', 0.0049); % k_CR1on, default = 0.0049

set(m1.Reactions(28).KineticLaw.Parameters(2), 'Value', 0.005); % k_CR1off, defaulat = 0.006

set(m1.Reactions(30).KineticLaw.Parameters(1), 'Value', 0.0049); % k_CR1Am_on

set(m1.Reactions(30).KineticLaw.Parameters(2), 'Value', 0.005); % k_CR1Am_off

set(m1.Reactions(18).KineticLaw.Parameters(1), 'Value', 0.0013); %k_trans default = 0.0013

set(m1.Reactions(19).KineticLaw.Parameters(1), 'Value', 0.0008); %k_transAm default = 8E-04

set(m1.Reactions(20).KineticLaw.Parameters(1), 'Value', 0.021); %k_mRNA_deg default = 0.021

set(m1.Reactions(21).KineticLaw.Parameters(1), 'Value', 0.002); %k_exp default = 0.002

set(m1.Reactions(29).KineticLaw.Parameters(1), 'Value', 0.0016); % k_trans_CR1 default = 0.0016

set(m1.Reactions(31).KineticLaw.Parameters(1), 'Value', 0.008); % k_transAm_CR1 default = 0.0011

set(m1.Reactions(22).KineticLaw.Parameters(1), 'Value', 1.0E-04); % kDinit

set(m1.Reactions(23).KineticLaw.Parameters(1), 'Value', 0.0019); % k_D_terminate

set(m1.Reactions(26).KineticLaw.Parameters(1), 'Value', 0.00142); %k_AHRsynth

set(m1.Reactions(27).KineticLaw.Parameters(1), 'Value', 0.0013); % k_ARNTsynth

%set(m1.Reactions(28), 'Active', false); % turn off CoReg binding

%set(m1.Reactions(30), 'Active', false); % turn of Am CoReg binding

%set(m1.Reactions(32), 'Active', false); % turn off other CoReg binding

%% nM old # molecules # molecules 10/08/13

% 1 0.0003 54

% 2 0.001 181 768

% 3 0.003 542 2300

% 4 0.01 1806 7662

% 5 0.03 5420 22995

% 6 0.1 18067 76651

% 7 0.3 54202 229960

% 8 1 180673 766520

% 9 3 542018 2299600

% 10 10 1806726 7665200

%% Chip time vector

%% Dosing

% Initialize variant used by the scan.

s1_TCDD_Values = [768, 2300, 7662, 22996, 76651, 229960, 766520, 2299600, 7665200];

scanVariant = sbiovariant('scanVariant','Tag','scanVariant');

runs = 100;

% Initialize output.

data = [];

t_rct = [];

%% Run scan.

sbioaccelerate(m1, cs);

for k1 = 1:length(s1_TCDD_Values)

% Set the variant's content (this removes the Content that was there in the previous run.)

%*************************************

set(scanVariant, 'Content', {'species', 'diffusionVol.TCDD', 'InitialAmount', s1_TCDD_Values(k1)});

%*************************************

k1

s1_TCDD_Values(k1)

sbioaccelerate(m1);

% Simulate the model.

for i = 1:runs

temp = sbiosimulate(m1, cs, scanVariant);

i

% Concatenate the output.

if isempty(data)

data = temp;

else

data = [data; temp]; %#ok<AGROW>

end %if

end %for

end

fprintf('done')

%% process by dose

Simp3_CoF3000_Oth2500_D1 = [];

for i = 1:runs

Simp3_CoF3000_Oth2500_D1 = [Simp3_CoF3000_Oth2500_D1; data(i)];

end;

fprintf(' D1');

[d1_Simp3_CoF3000_Oth2500.time, d1_Simp3_CoF3000_Oth2500.avg, d1_Simp3_CoF3000_Oth2500.var] = sbioensemblestats(Simp3_CoF3000_Oth2500_D1);

Simp3_CoF3000_Oth2500_D2 = [];

for i = runs+1:2*runs

Simp3_CoF3000_Oth2500_D2 = [Simp3_CoF3000_Oth2500_D2; data(i)];

end;

fprintf(' D2');

[d2_Simp3_CoF3000_Oth2500.time, d2_Simp3_CoF3000_Oth2500.avg, d2_Simp3_CoF3000_Oth2500.var] = sbioensemblestats(Simp3_CoF3000_Oth2500_D2);

Simp3_CoF3000_Oth2500_D3 = [];

for i = 2*runs+1:3*runs

Simp3_CoF3000_Oth2500_D3 = [Simp3_CoF3000_Oth2500_D3; data(i)];

end;

fprintf(' D3');

[d3_Simp3_CoF3000_Oth2500.time, d3_Simp3_CoF3000_Oth2500.avg, d3_Simp3_CoF3000_Oth2500.var] = sbioensemblestats(Simp3_CoF3000_Oth2500_D3);

Simp3_CoF3000_Oth2500_D4 = [];

for i = 3*runs+1:4*runs

Simp3_CoF3000_Oth2500_D4 = [Simp3_CoF3000_Oth2500_D4; data(i)];

end;

fprintf(' D4');

[d4_Simp3_CoF3000_Oth2500.time, d4_Simp3_CoF3000_Oth2500.avg, d4_Simp3_CoF3000_Oth2500.var] = sbioensemblestats(Simp3_CoF3000_Oth2500_D4);

Simp3_CoF3000_Oth2500_D5 = [];

for i = 4*runs+1:5*runs

Simp3_CoF3000_Oth2500_D5 = [Simp3_CoF3000_Oth2500_D5; data(i)];

end;

fprintf(' D5');

[d5_Simp3_CoF3000_Oth2500.time, d5_Simp3_CoF3000_Oth2500.avg, d5_Simp3_CoF3000_Oth2500.var] = sbioensemblestats(Simp3_CoF3000_Oth2500_D5);

Simp3_CoF3000_Oth2500_D6 = [];

for i = 5*runs+1:6*runs

Simp3_CoF3000_Oth2500_D6 = [Simp3_CoF3000_Oth2500_D6; data(i)];

end;

fprintf(' D6');

[d6_Simp3_CoF3000_Oth2500.time, d6_Simp3_CoF3000_Oth2500.avg, d6_Simp3_CoF3000_Oth2500.var] = sbioensemblestats(Simp3_CoF3000_Oth2500_D6);

Simp3_CoF3000_Oth2500_D7 = [];

for i = 6*runs+1:7*runs

Simp3_CoF3000_Oth2500_D7 = [Simp3_CoF3000_Oth2500_D7; data(i)];

end;

fprintf(' D7');

[d7_Simp3_CoF3000_Oth2500.time, d7_Simp3_CoF3000_Oth2500.avg, d7_Simp3_CoF3000_Oth2500.var] = sbioensemblestats(Simp3_CoF3000_Oth2500_D7);

Simp3_CoF3000_Oth2500_D8 = [];

for i = 7*runs+1:8*runs

Simp3_CoF3000_Oth2500_D8 = [Simp3_CoF3000_Oth2500_D8; data(i)];

end;

fprintf(' D8');

[d8_Simp3_CoF3000_Oth2500.time, d8_Simp3_CoF3000_Oth2500.avg, d8_Simp3_CoF3000_Oth2500.var] = sbioensemblestats(Simp3_CoF3000_Oth2500_D8);

Simp3_CoF3000_Oth2500_D9 = [];

for i = 8*runs+1:9*runs

Simp3_CoF3000_Oth2500_D9 = [Simp3_CoF3000_Oth2500_D9; data(i)];

end;

fprintf(' D9');

[d9_Simp3_CoF3000_Oth2500.time, d9_Simp3_CoF3000_Oth2500.avg, d9_Simp3_CoF3000_Oth2500.var] = sbioensemblestats(Simp3_CoF3000_Oth2500_D9);

d1_Simp3_CoF3000_Oth2500.avg(length(d1_Simp3_CoF3000_Oth2500.avg(:,17)),17)

d2_Simp3_CoF3000_Oth2500.avg(length(d2_Simp3_CoF3000_Oth2500.avg(:,17)),17)

d3_Simp3_CoF3000_Oth2500.avg(length(d3_Simp3_CoF3000_Oth2500.avg(:,17)),17)

d4_Simp3_CoF3000_Oth2500.avg(length(d4_Simp3_CoF3000_Oth2500.avg(:,17)),17)

d5_Simp3_CoF3000_Oth2500.avg(length(d5_Simp3_CoF3000_Oth2500.avg(:,17)),17)

d6_Simp3_CoF3000_Oth2500.avg(length(d6_Simp3_CoF3000_Oth2500.avg(:,17)),17)

d7_Simp3_CoF3000_Oth2500.avg(length(d7_Simp3_CoF3000_Oth2500.avg(:,17)),17)

d8_Simp3_CoF3000_Oth2500.avg(length(d8_Simp3_CoF3000_Oth2500.avg(:,17)),17)

d9_Simp3_CoF3000_Oth2500.avg(length(d9_Simp3_CoF3000_Oth2500.avg(:,17)),17)

%% AA_CoF CYPDRE_T CYPDRE_T_Pol CYP_init CYP_init_Am mRNA_t mRNA ARNT

%figure;

subplot(5,2,1), plot(d1_Simp3_CoF3000_Oth2500.time, d1_Simp3_CoF3000_Oth2500.avg(:,1), '-bl'); hold on;

subplot(5,2,1), plot(d2_Simp3_CoF3000_Oth2500.time, d2_Simp3_CoF3000_Oth2500.avg(:,1), '-m'); hold on;

subplot(5,2,1), plot(d3_Simp3_CoF3000_Oth2500.time, d3_Simp3_CoF3000_Oth2500.avg(:,1), '-c'); hold on;

subplot(5,2,1), plot(d4_Simp3_CoF3000_Oth2500.time, d4_Simp3_CoF3000_Oth2500.avg(:,1), '-b'); hold on;

subplot(5,2,1), plot(d5_Simp3_CoF3000_Oth2500.time, d5_Simp3_CoF3000_Oth2500.avg(:,1), '-r'); hold on;

subplot(5,2,1), plot(d6_Simp3_CoF3000_Oth2500.time, d6_Simp3_CoF3000_Oth2500.avg(:,1), '-r'); hold on;

subplot(5,2,1), plot(d7_Simp3_CoF3000_Oth2500.time, d7_Simp3_CoF3000_Oth2500.avg(:,1), '-r'); hold on;

subplot(5,2,1), plot(d8_Simp3_CoF3000_Oth2500.time, d8_Simp3_CoF3000_Oth2500.avg(:,1), '-r'); hold on;

subplot(5,2,1), plot(d9_Simp3_CoF3000_Oth2500.time, d9_Simp3_CoF3000_Oth2500.avg(:,1), '-r'); hold on;

legend('AHR');

subplot(5,2,2), plot(d1_Simp3_CoF3000_Oth2500.time, d1_Simp3_CoF3000_Oth2500.avg(:,2), '-b'); hold on;

subplot(5,2,2), plot(d2_Simp3_CoF3000_Oth2500.time, d2_Simp3_CoF3000_Oth2500.avg(:,2), '-m'); hold on;

subplot(5,2,2), plot(d3_Simp3_CoF3000_Oth2500.time, d3_Simp3_CoF3000_Oth2500.avg(:,2), '-c'); hold on;

subplot(5,2,2), plot(d4_Simp3_CoF3000_Oth2500.time, d4_Simp3_CoF3000_Oth2500.avg(:,2), '-b'); hold on;

subplot(5,2,2), plot(d5_Simp3_CoF3000_Oth2500.time, d5_Simp3_CoF3000_Oth2500.avg(:,2), '-r'); hold on;

subplot(5,2,2), plot(d6_Simp3_CoF3000_Oth2500.time, d6_Simp3_CoF3000_Oth2500.avg(:,2), '-r'); hold on;

subplot(5,2,2), plot(d7_Simp3_CoF3000_Oth2500.time, d7_Simp3_CoF3000_Oth2500.avg(:,2), '-r'); hold on;

subplot(5,2,2), plot(d8_Simp3_CoF3000_Oth2500.time, d8_Simp3_CoF3000_Oth2500.avg(:,2), '-r'); hold on;

subplot(5,2,2), plot(d9_Simp3_CoF3000_Oth2500.time, d9_Simp3_CoF3000_Oth2500.avg(:,2), '-r'); hold on;

legend('AHR-TCDD');

subplot(5,2,3), plot(d1_Simp3_CoF3000_Oth2500.time, d1_Simp3_CoF3000_Oth2500.avg(:,6), '-bl'); hold on;

subplot(5,2,3), plot(d2_Simp3_CoF3000_Oth2500.time, d2_Simp3_CoF3000_Oth2500.avg(:,6), '-m'); hold on;

subplot(5,2,3), plot(d3_Simp3_CoF3000_Oth2500.time, d3_Simp3_CoF3000_Oth2500.avg(:,6), '-c'); hold on;

subplot(5,2,3), plot(d4_Simp3_CoF3000_Oth2500.time, d4_Simp3_CoF3000_Oth2500.avg(:,6), '-b'); hold on;

subplot(5,2,3), plot(d5_Simp3_CoF3000_Oth2500.time, d5_Simp3_CoF3000_Oth2500.avg(:,6), '-r'); hold on;

subplot(5,2,3), plot(d6_Simp3_CoF3000_Oth2500.time, d6_Simp3_CoF3000_Oth2500.avg(:,6), '-r'); hold on;

subplot(5,2,3), plot(d7_Simp3_CoF3000_Oth2500.time, d7_Simp3_CoF3000_Oth2500.avg(:,6), '-r'); hold on;

subplot(5,2,3), plot(d9_Simp3_CoF3000_Oth2500.time, d9_Simp3_CoF3000_Oth2500.avg(:,6), '-r'); hold on;

legend('AHR-ARNT');

subplot(5,2,4), plot(d1_Simp3_CoF3000_Oth2500.time, d1_Simp3_CoF3000_Oth2500.avg(:,9), '-bl'); hold on;

subplot(5,2,4), plot(d2_Simp3_CoF3000_Oth2500.time, d2_Simp3_CoF3000_Oth2500.avg(:,9), '-m'); hold on;

subplot(5,2,4), plot(d3_Simp3_CoF3000_Oth2500.time, d3_Simp3_CoF3000_Oth2500.avg(:,9), '-c'); hold on;

subplot(5,2,4), plot(d4_Simp3_CoF3000_Oth2500.time, d4_Simp3_CoF3000_Oth2500.avg(:,9), '-b'); hold on;

subplot(5,2,4), plot(d5_Simp3_CoF3000_Oth2500.time, d5_Simp3_CoF3000_Oth2500.avg(:,9), '-r'); hold on;

subplot(5,2,4), plot(d6_Simp3_CoF3000_Oth2500.time, d6_Simp3_CoF3000_Oth2500.avg(:,9), '-r'); hold on;

subplot(5,2,4), plot(d7_Simp3_CoF3000_Oth2500.time, d7_Simp3_CoF3000_Oth2500.avg(:,9), '-r'); hold on;

subplot(5,2,4), plot(d8_Simp3_CoF3000_Oth2500.time, d8_Simp3_CoF3000_Oth2500.avg(:,9), '-r'); hold on;

subplot(5,2,4), plot(d9_Simp3_CoF3000_Oth2500.time, d9_Simp3_CoF3000_Oth2500.avg(:,9), '-r'); hold on;

legend('AA-CoF');

subplot(5,2,5), plot(d1_Simp3_CoF3000_Oth2500.time, d1_Simp3_CoF3000_Oth2500.avg(:,13), '-bl'); hold on;

subplot(5,2,5), plot(d2_Simp3_CoF3000_Oth2500.time, d2_Simp3_CoF3000_Oth2500.avg(:,13), '-m'); hold on;

subplot(5,2,5), plot(d3_Simp3_CoF3000_Oth2500.time, d3_Simp3_CoF3000_Oth2500.avg(:,13), '-c'); hold on;

subplot(5,2,5), plot(d4_Simp3_CoF3000_Oth2500.time, d4_Simp3_CoF3000_Oth2500.avg(:,13), '-b'); hold on;

subplot(5,2,5), plot(d5_Simp3_CoF3000_Oth2500.time, d5_Simp3_CoF3000_Oth2500.avg(:,13), '-r'); hold on;

subplot(5,2,5), plot(d6_Simp3_CoF3000_Oth2500.time, d6_Simp3_CoF3000_Oth2500.avg(:,13), '-r'); hold on;

subplot(5,2,5), plot(d7_Simp3_CoF3000_Oth2500.time, d7_Simp3_CoF3000_Oth2500.avg(:,13), '-r'); hold on;

subplot(5,2,5), plot(d8_Simp3_CoF3000_Oth2500.time, d8_Simp3_CoF3000_Oth2500.avg(:,13), '-r'); hold on;

subplot(5,2,5), plot(d9_Simp3_CoF3000_Oth2500.time, d9_Simp3_CoF3000_Oth2500.avg(:,13), '-r'); hold on;

legend('CYPDRE-T');

subplot(5,2,6), plot(d1_Simp3_CoF3000_Oth2500.time, d1_Simp3_CoF3000_Oth2500.avg(:,16), '-bl'); hold on;

subplot(5,2,6), plot(d2_Simp3_CoF3000_Oth2500.time, d2_Simp3_CoF3000_Oth2500.avg(:,16), '-m'); hold on;

subplot(5,2,6), plot(d3_Simp3_CoF3000_Oth2500.time, d3_Simp3_CoF3000_Oth2500.avg(:,16), '-c'); hold on;

subplot(5,2,6), plot(d4_Simp3_CoF3000_Oth2500.time, d4_Simp3_CoF3000_Oth2500.avg(:,16), '-b'); hold on;

subplot(5,2,6), plot(d5_Simp3_CoF3000_Oth2500.time, d5_Simp3_CoF3000_Oth2500.avg(:,16), '-r'); hold on;

subplot(5,2,6), plot(d6_Simp3_CoF3000_Oth2500.time, d6_Simp3_CoF3000_Oth2500.avg(:,16), '-r'); hold on;

subplot(5,2,6), plot(d7_Simp3_CoF3000_Oth2500.time, d7_Simp3_CoF3000_Oth2500.avg(:,16), '-r'); hold on;

subplot(5,2,6), plot(d8_Simp3_CoF3000_Oth2500.time, d8_Simp3_CoF3000_Oth2500.avg(:,16), '-r'); hold on;

subplot(5,2,6), plot(d9_Simp3_CoF3000_Oth2500.time, d9_Simp3_CoF3000_Oth2500.avg(:,16), '-r'); hold on;

legend('CYPDRE-T-Pol');

subplot(5,2,7), plot(d1_Simp3_CoF3000_Oth2500.time, d1_Simp3_CoF3000_Oth2500.avg(:,19), d1_Simp3_CoF3000_Oth2500.time, d1_Simp3_CoF3000_Oth2500.avg(:,20));

subplot(5,2,7), plot(d2_Simp3_CoF3000_Oth2500.time, d2_Simp3_CoF3000_Oth2500.avg(:,19), d2_Simp3_CoF3000_Oth2500.time, d2_Simp3_CoF3000_Oth2500.avg(:,20));

subplot(5,2,7), plot(d3_Simp3_CoF3000_Oth2500.time, d3_Simp3_CoF3000_Oth2500.avg(:,19), d3_Simp3_CoF3000_Oth2500.time, d3_Simp3_CoF3000_Oth2500.avg(:,20));

subplot(5,2,7), plot(d4_Simp3_CoF3000_Oth2500.time, d4_Simp3_CoF3000_Oth2500.avg(:,19), d4_Simp3_CoF3000_Oth2500.time, d4_Simp3_CoF3000_Oth2500.avg(:,20));

subplot(5,2,7), plot(d5_Simp3_CoF3000_Oth2500.time, d5_Simp3_CoF3000_Oth2500.avg(:,19), d5_Simp3_CoF3000_Oth2500.time, d5_Simp3_CoF3000_Oth2500.avg(:,20));

subplot(5,2,7), plot(d6_Simp3_CoF3000_Oth2500.time, d6_Simp3_CoF3000_Oth2500.avg(:,19), d6_Simp3_CoF3000_Oth2500.time, d6_Simp3_CoF3000_Oth2500.avg(:,20));

subplot(5,2,7), plot(d7_Simp3_CoF3000_Oth2500.time, d7_Simp3_CoF3000_Oth2500.avg(:,19), d7_Simp3_CoF3000_Oth2500.time, d7_Simp3_CoF3000_Oth2500.avg(:,20));

subplot(5,2,7), plot(d8_Simp3_CoF3000_Oth2500.time, d8_Simp3_CoF3000_Oth2500.avg(:,19), d8_Simp3_CoF3000_Oth2500.time, d8_Simp3_CoF3000_Oth2500.avg(:,20));

subplot(5,2,7), plot(d9_Simp3_CoF3000_Oth2500.time, d9_Simp3_CoF3000_Oth2500.avg(:,19), d9_Simp3_CoF3000_Oth2500.time, d9_Simp3_CoF3000_Oth2500.avg(:,20));

legend('CYP-init','CYP-Am-init');

subplot(5,2,8), plot(d1_Simp3_CoF3000_Oth2500.time, d1_Simp3_CoF3000_Oth2500.avg(:,17), '-bl'); hold on;

subplot(5,2,8), plot(d2_Simp3_CoF3000_Oth2500.time, d2_Simp3_CoF3000_Oth2500.avg(:,17), '-m'); hold on;

subplot(5,2,8), plot(d3_Simp3_CoF3000_Oth2500.time, d3_Simp3_CoF3000_Oth2500.avg(:,17), '-c'); hold on;

subplot(5,2,8), plot(d4_Simp3_CoF3000_Oth2500.time, d4_Simp3_CoF3000_Oth2500.avg(:,17), '-b'); hold on;

subplot(5,2,8), plot(d5_Simp3_CoF3000_Oth2500.time, d5_Simp3_CoF3000_Oth2500.avg(:,17), '-r'); hold on;

subplot(5,2,8), plot(d6_Simp3_CoF3000_Oth2500.time, d6_Simp3_CoF3000_Oth2500.avg(:,17), '-r'); hold on;

subplot(5,2,8), plot(d7_Simp3_CoF3000_Oth2500.time, d7_Simp3_CoF3000_Oth2500.avg(:,17), '-r'); hold on;

subplot(5,2,8), plot(d8_Simp3_CoF3000_Oth2500.time, d8_Simp3_CoF3000_Oth2500.avg(:,17), '-r'); hold on;

subplot(5,2,8), plot(d9_Simp3_CoF3000_Oth2500.time, d9_Simp3_CoF3000_Oth2500.avg(:,17), '-r'); hold on;

legend('mRNA');

subplot(5,2,9), plot(d1_Simp3_CoF3000_Oth2500.time, d1_Simp3_CoF3000_Oth2500.avg(:,18), '-bl'); hold on;

subplot(5,2,9), plot(d2_Simp3_CoF3000_Oth2500.time, d2_Simp3_CoF3000_Oth2500.avg(:,18), '-m'); hold on;

subplot(5,2,9), plot(d3_Simp3_CoF3000_Oth2500.time, d3_Simp3_CoF3000_Oth2500.avg(:,18), '-c'); hold on;

subplot(5,2,9), plot(d4_Simp3_CoF3000_Oth2500.time, d4_Simp3_CoF3000_Oth2500.avg(:,18), '-b'); hold on;

subplot(5,2,9), plot(d5_Simp3_CoF3000_Oth2500.time, d5_Simp3_CoF3000_Oth2500.avg(:,18), '-r'); hold on;

subplot(5,2,9), plot(d6_Simp3_CoF3000_Oth2500.time, d6_Simp3_CoF3000_Oth2500.avg(:,18), '-r'); hold on;

subplot(5,2,9), plot(d7_Simp3_CoF3000_Oth2500.time, d7_Simp3_CoF3000_Oth2500.avg(:,18), '-r'); hold on;

subplot(5,2,9), plot(d8_Simp3_CoF3000_Oth2500.time, d8_Simp3_CoF3000_Oth2500.avg(:,18), '-r'); hold on;

subplot(5,2,9), plot(d9_Simp3_CoF3000_Oth2500.time, d9_Simp3_CoF3000_Oth2500.avg(:,18), '-r'); hold on;

legend('DRE-T-Pol');

subplot(5,2,10), plot(d1_Simp3_CoF3000_Oth2500.time, d1_Simp3_CoF3000_Oth2500.avg(:,22), d1_Simp3_CoF3000_Oth2500.time, d1_Simp3_CoF3000_Oth2500.avg(:,23));

subplot(5,2,10), plot(d2_Simp3_CoF3000_Oth2500.time, d2_Simp3_CoF3000_Oth2500.avg(:,22), d2_Simp3_CoF3000_Oth2500.time, d2_Simp3_CoF3000_Oth2500.avg(:,23));

subplot(5,2,10), plot(d3_Simp3_CoF3000_Oth2500.time, d3_Simp3_CoF3000_Oth2500.avg(:,22), d3_Simp3_CoF3000_Oth2500.time, d3_Simp3_CoF3000_Oth2500.avg(:,23));

subplot(5,2,10), plot(d4_Simp3_CoF3000_Oth2500.time, d4_Simp3_CoF3000_Oth2500.avg(:,22), d4_Simp3_CoF3000_Oth2500.time, d4_Simp3_CoF3000_Oth2500.avg(:,23));

subplot(5,2,10), plot(d5_Simp3_CoF3000_Oth2500.time, d5_Simp3_CoF3000_Oth2500.avg(:,22), d5_Simp3_CoF3000_Oth2500.time, d5_Simp3_CoF3000_Oth2500.avg(:,23));

subplot(5,2,10), plot(d6_Simp3_CoF3000_Oth2500.time, d6_Simp3_CoF3000_Oth2500.avg(:,22), d6_Simp3_CoF3000_Oth2500.time, d6_Simp3_CoF3000_Oth2500.avg(:,23));

subplot(5,2,10), plot(d7_Simp3_CoF3000_Oth2500.time, d7_Simp3_CoF3000_Oth2500.avg(:,22), d7_Simp3_CoF3000_Oth2500.time, d7_Simp3_CoF3000_Oth2500.avg(:,23));

subplot(5,2,10), plot(d8_Simp3_CoF3000_Oth2500.time, d8_Simp3_CoF3000_Oth2500.avg(:,22), d8_Simp3_CoF3000_Oth2500.time, d8_Simp3_CoF3000_Oth2500.avg(:,23));

subplot(5,2,10), plot(d9_Simp3_CoF3000_Oth2500.time, d9_Simp3_CoF3000_Oth2500.avg(:,22), d9_Simp3_CoF3000_Oth2500.time, d9_Simp3_CoF3000_Oth2500.avg(:,23));

legend('DRE-init', 'DRE-Am-init')

hold off;

**Code B. SBML Code for the model**

<?xml version="1.0" encoding="UTF-8"?>

<sbml xmlns="http://www.sbml.org/sbml/level2/version4" level="2" version="4">

<annotation>

<SimBiology xmlns="http://www.mathworks.com">

<Version Major="4" Minor="2" Point="0"/>

</SimBiology>

</annotation>

<model id="mw96adee4b_018a_478d_a242_68c96efadb60" name="TCDD_simp3_011514">

<notes>

<body xmlns="http://www.w3.org/1999/xhtml">for a volume of 3E-10 L as the outside diffusion volume, this table gives nM conc in molecules

nM molecules

0.001 181

0.003 542

0.01 1806

0.03 5420

0.1 18067

0.3 54202

1 180673

3 542018

10 1806726

30 5420177

100 18067257

</body>

</notes>

<listOfUnitDefinitions>

<unitDefinition id="MWBUILTINUNIT_liter" name="liter">

<listOfUnits>

<unit kind="metre" exponent="3"/>

<unit kind="dimensionless" multiplier="0.001"/>

</listOfUnits>

</unitDefinition>

<unitDefinition id="MWBUILTINUNIT_molecule" name="molecule">

<listOfUnits>

<unit kind="mole" multiplier="1.66053872801495e-24"/>

</listOfUnits>

</unitDefinition>

<unitDefinition id="MWDERIVEDUNIT_1__second" name="1/second">

<listOfUnits>

<unit kind="second" exponent="-1"/>

<unit kind="dimensionless"/>

</listOfUnits>

</unitDefinition>

<unitDefinition id="MWDERIVEDUNIT_1__molecule_second" name="1/(molecule*second)">

<listOfUnits>

<unit kind="mole" exponent="-1"/>

<unit kind="second" exponent="-1"/>

<unit kind="dimensionless" multiplier="6.02214199e+23"/>

</listOfUnits>

</unitDefinition>

<unitDefinition id="MWDERIVEDUNIT_molecule__second" name="molecule/second">

<listOfUnits>

<unit kind="mole"/>

<unit kind="second" exponent="-1"/>

<unit kind="dimensionless" multiplier="1.66053872801495e-24"/>

</listOfUnits>

</unitDefinition>

</listOfUnitDefinitions>

<listOfCompartments>

<compartment id="mw386d9a9b_be58_402d_a282_d93fdc28c9ff" name="diffusionVol" size="3e-10" units="MWBUILTINUNIT_liter"/>

<compartment id="mw197f41b7_d6bc_415e_b3db_d2a8404137a3" name="cell" size="1.25e-12" units="MWBUILTINUNIT_liter" outside="mw386d9a9b_be58_402d_a282_d93fdc28c9ff"/>

<compartment id="mw3b65cf96_fe6e_4e70_bf87_ecf6ea481558" name="nucleus" size="5.1e-13" units="MWBUILTINUNIT_liter" outside="mw197f41b7_d6bc_415e_b3db_d2a8404137a3"/>

<compartment id="mwcfb998db_c3a6_4616_aaa4_affdabbfd7ae" name="transcription" size="2e-14" units="MWBUILTINUNIT_liter" outside="mw3b65cf96_fe6e_4e70_bf87_ecf6ea481558"/>

</listOfCompartments>

<listOfSpecies>

<species id="mwf0ec6eb5_aaa2_47a8_a217_043de1df28f2" name="CYP_init" compartment="mwcfb998db_c3a6_4616_aaa4_affdabbfd7ae" initialAmount="0" substanceUnits="MWBUILTINUNIT_molecule" hasOnlySubstanceUnits="true"/>

<species id="mw97f7ade5_32dd_455a_a70f_bd86fcf4b24e" name="CYP_init_Am" compartment="mwcfb998db_c3a6_4616_aaa4_affdabbfd7ae" initialAmount="0" substanceUnits="MWBUILTINUNIT_molecule" hasOnlySubstanceUnits="true"/>

<species id="mwf105777a_6633_4581_b5f6_a44407454a24" name="mRNA_t" compartment="mwcfb998db_c3a6_4616_aaa4_affdabbfd7ae" initialAmount="0" substanceUnits="MWBUILTINUNIT_molecule" hasOnlySubstanceUnits="true"/>

<species id="mwdeca1e63_9abf_4025_90ce_0f9d548e2dc3" name="DRE_init" compartment="mwcfb998db_c3a6_4616_aaa4_affdabbfd7ae" initialAmount="0" substanceUnits="MWBUILTINUNIT_molecule" hasOnlySubstanceUnits="true"/>

<species id="mwe9d69afa_bb35_4453_bd01_c3db9dc6acea" name="DRE_init_Am" compartment="mwcfb998db_c3a6_4616_aaa4_affdabbfd7ae" initialAmount="0" substanceUnits="MWBUILTINUNIT_molecule" hasOnlySubstanceUnits="true"/>

<species id="mw21412c31_04c5_44ef_9f26_8cf3ae3b1ddd" name="CoReg1" compartment="mwcfb998db_c3a6_4616_aaa4_affdabbfd7ae" initialAmount="100" substanceUnits="MWBUILTINUNIT_molecule" hasOnlySubstanceUnits="true"/>

<species id="mwf15006e7_b584_4348_90d9_9498e0fe36ac" name="CYP_CoReg1" compartment="mwcfb998db_c3a6_4616_aaa4_affdabbfd7ae" initialAmount="0" substanceUnits="MWBUILTINUNIT_molecule" hasOnlySubstanceUnits="true"/>

<species id="mw71c5a552_df36_4e17_87f2_3896da9028dc" name="CYP_Am_CoReg1" compartment="mwcfb998db_c3a6_4616_aaa4_affdabbfd7ae" initialAmount="0" substanceUnits="MWBUILTINUNIT_molecule" hasOnlySubstanceUnits="true"/>

<species id="mwa662fddd_da79_454e_ab3d_4068859a29ba" name="CoR_Other" compartment="mwcfb998db_c3a6_4616_aaa4_affdabbfd7ae" initialAmount="1000" substanceUnits="MWBUILTINUNIT_molecule" hasOnlySubstanceUnits="true"/>

<species id="mw231f14b3_d84e_4d35_aac3_eaac7f318a3b" name="Bound_CoR" compartment="mwcfb998db_c3a6_4616_aaa4_affdabbfd7ae" initialAmount="10" substanceUnits="MWBUILTINUNIT_molecule" hasOnlySubstanceUnits="true"/>

<species id="mwd9296a48_0796_4faa_87c8_e55eb6aa96bc" name="AHR-TCDD" compartment="mw3b65cf96_fe6e_4e70_bf87_ecf6ea481558" initialAmount="0" substanceUnits="MWBUILTINUNIT_molecule" hasOnlySubstanceUnits="true"/>

<species id="mw4aba41df_adfc_4ccc_b5e3_7a838f8abed1" name="ARNT" compartment="mw3b65cf96_fe6e_4e70_bf87_ecf6ea481558" initialAmount="3686" substanceUnits="MWBUILTINUNIT_molecule" hasOnlySubstanceUnits="true"/>

<species id="mwf9e37e2b_58fb_42b3_ae54_34c6e14f0084" name="AHR_ARNT" compartment="mw3b65cf96_fe6e_4e70_bf87_ecf6ea481558" initialAmount="0" substanceUnits="MWBUILTINUNIT_molecule" hasOnlySubstanceUnits="true"/>

<species id="mwfd381d44_fd59_4865_879d_f0676e15b3a2" name="CoF" compartment="mw3b65cf96_fe6e_4e70_bf87_ecf6ea481558" initialAmount="1500" substanceUnits="MWBUILTINUNIT_molecule" hasOnlySubstanceUnits="true"/>

<species id="mw78afaec6_2a0b_4290_a12b_d967e9d89d31" name="Other" compartment="mw3b65cf96_fe6e_4e70_bf87_ecf6ea481558" initialAmount="1535" substanceUnits="MWBUILTINUNIT_molecule" hasOnlySubstanceUnits="true"/>

<species id="mw661625ec_78bf_4971_b983_e2b0553aa688" name="AA_CoF" compartment="mw3b65cf96_fe6e_4e70_bf87_ecf6ea481558" initialAmount="0" substanceUnits="MWBUILTINUNIT_molecule" hasOnlySubstanceUnits="true"/>

<species id="mwc646630d_524d_4b59_890b_fcdd19f0041f" name="Other_CoF" compartment="mw3b65cf96_fe6e_4e70_bf87_ecf6ea481558" initialAmount="0" substanceUnits="MWBUILTINUNIT_molecule" hasOnlySubstanceUnits="true"/>

<species id="mwd841ce40_b3fe_4388_b462_0e3b09be524d" name="CYPDRE" compartment="mw3b65cf96_fe6e_4e70_bf87_ecf6ea481558" initialAmount="4" substanceUnits="MWBUILTINUNIT_molecule" hasOnlySubstanceUnits="true"/>

<species id="mw9bea94b6_388a_46f0_9e30_8df1c202c739" name="DRE" compartment="mw3b65cf96_fe6e_4e70_bf87_ecf6ea481558" initialAmount="470" substanceUnits="MWBUILTINUNIT_molecule" hasOnlySubstanceUnits="true"/>

<species id="mw455a157e_ff7c_426b_a943_e1b8cd1cd808" name="CYPDRE_T" compartment="mw3b65cf96_fe6e_4e70_bf87_ecf6ea481558" initialAmount="0" substanceUnits="MWBUILTINUNIT_molecule" hasOnlySubstanceUnits="true"/>

<species id="mw41e86661_220e_4030_8f3e_eba2564e78e3" name="DRE_T" compartment="mw3b65cf96_fe6e_4e70_bf87_ecf6ea481558" initialAmount="0" substanceUnits="MWBUILTINUNIT_molecule" hasOnlySubstanceUnits="true"/>

<species id="mw1f0ddd1b_48a1_464a_951c_3b29d2b19572" name="RNA_Pol" compartment="mw3b65cf96_fe6e_4e70_bf87_ecf6ea481558" initialAmount="4000" substanceUnits="MWBUILTINUNIT_molecule" hasOnlySubstanceUnits="true"/>

<species id="mw2973f81c_7b29_409d_b2ac_dc0be0780ad2" name="CYPDRE_T_Pol" compartment="mw3b65cf96_fe6e_4e70_bf87_ecf6ea481558" initialAmount="0" substanceUnits="MWBUILTINUNIT_molecule" hasOnlySubstanceUnits="true"/>

<species id="mwae4b2487_0fa8_4a41_ad45_2ae11e66fdc5" name="mRNA" compartment="mw3b65cf96_fe6e_4e70_bf87_ecf6ea481558" initialAmount="1" substanceUnits="MWBUILTINUNIT_molecule" hasOnlySubstanceUnits="true"/>

<species id="mwec8f418e_d8a8_472e_8162_04976fca96ea" name="DRE_T_Pol" compartment="mw3b65cf96_fe6e_4e70_bf87_ecf6ea481558" initialAmount="0" substanceUnits="MWBUILTINUNIT_molecule" hasOnlySubstanceUnits="true"/>

<species id="mw9cfde077_d7d5_406d_b8ab_2e87f7f4f091" name="TCDD" compartment="mw197f41b7_d6bc_415e_b3db_d2a8404137a3" initialAmount="0" substanceUnits="MWBUILTINUNIT_molecule" hasOnlySubstanceUnits="true"/>

<species id="mwe0a75c34_5ffa_48fe_830f_bfcf5b6e61c5" name="AHR" compartment="mw197f41b7_d6bc_415e_b3db_d2a8404137a3" initialAmount="8333" substanceUnits="MWBUILTINUNIT_molecule" hasOnlySubstanceUnits="true"/>

<species id="mwe70acfc0_34b6_4157_9905_e73cd58e595e" name="TCDD-AHR" compartment="mw197f41b7_d6bc_415e_b3db_d2a8404137a3" initialAmount="0" substanceUnits="MWBUILTINUNIT_molecule" hasOnlySubstanceUnits="true"/>

<species id="mwbde73437_babc_4972_a83b_2093be8f438a" name="TCDD" compartment="mw386d9a9b_be58_402d_a282_d93fdc28c9ff" initialAmount="229960" substanceUnits="MWBUILTINUNIT_molecule" hasOnlySubstanceUnits="true" constant="true"/>

</listOfSpecies>

<listOfReactions>

<reaction id="mw957265d2_4055_4bcd_b78c_d841a1f6bc32" name="Diffusion into cell" fast="false">

<listOfReactants>

<speciesReference species="mwbde73437_babc_4972_a83b_2093be8f438a"/>

</listOfReactants>

<listOfProducts>

<speciesReference species="mw9cfde077_d7d5_406d_b8ab_2e87f7f4f091"/>

</listOfProducts>

<kineticLaw>

<math xmlns="http://www.w3.org/1998/Math/MathML">

<apply>

<minus/>

<apply>

<times/>

<ci> mw2ce9c06a_9de5_46a2_a22f_274ea30bf2f7 </ci>

<ci> mwbde73437_babc_4972_a83b_2093be8f438a </ci>

</apply>

<apply>

<times/>

<ci> mw2ce9c06a_9de5_46a2_a22f_274ea30bf2f7 </ci>

<ci> mw9cfde077_d7d5_406d_b8ab_2e87f7f4f091 </ci>

</apply>

</apply>

</math>

<listOfParameters>

<parameter id="mw2ce9c06a_9de5_46a2_a22f_274ea30bf2f7" name="kdiff" value="8e-07" units="MWDERIVEDUNIT_1__second"/>

</listOfParameters>

</kineticLaw>

</reaction>

<reaction id="mw57722f22_bac0_4354_b4aa_b4c5ed136c82" name="TCDD binding to AHR" fast="false">

<listOfReactants>

<speciesReference species="mw9cfde077_d7d5_406d_b8ab_2e87f7f4f091"/>

<speciesReference species="mwe0a75c34_5ffa_48fe_830f_bfcf5b6e61c5"/>

</listOfReactants>

<listOfProducts>

<speciesReference species="mwe70acfc0_34b6_4157_9905_e73cd58e595e"/>

</listOfProducts>

<kineticLaw>

<math xmlns="http://www.w3.org/1998/Math/MathML">

<apply>

<minus/>

<apply>

<times/>

<ci> mwa18f9035_e0f8_4f2d_a1c7_f663f86f78ca </ci>

<ci> mw9cfde077_d7d5_406d_b8ab_2e87f7f4f091 </ci>

<ci> mwe0a75c34_5ffa_48fe_830f_bfcf5b6e61c5 </ci>

</apply>

<apply>

<times/>

<ci> mw80a9bb10_fbd9_4104_baab_ce86ea194033 </ci>

<ci> mwe70acfc0_34b6_4157_9905_e73cd58e595e </ci>

</apply>

</apply>

</math>

<listOfParameters>

<parameter id="mwa18f9035_e0f8_4f2d_a1c7_f663f86f78ca" name="kf1" value="5.3e-07" units="MWDERIVEDUNIT_1__molecule_second"/>

<parameter id="mw80a9bb10_fbd9_4104_baab_ce86ea194033" name="kb1" value="0.002" units="MWDERIVEDUNIT_1__second"/>

</listOfParameters>

</kineticLaw>

</reaction>

<reaction id="mw32d40a83_6e23_453b_a1d3_452f7cd370ba" name="Translocation" fast="false">

<notes>

<body xmlns="http://www.w3.org/1999/xhtml">From Fig. 10 in Richter et al 2001 Arch Biochem Biophys 398:207</body>

</notes>

<listOfReactants>

<speciesReference species="mwe70acfc0_34b6_4157_9905_e73cd58e595e"/>

</listOfReactants>

<listOfProducts>

<speciesReference species="mwd9296a48_0796_4faa_87c8_e55eb6aa96bc"/>

</listOfProducts>

<kineticLaw>

<math xmlns="http://www.w3.org/1998/Math/MathML">

<apply>

<minus/>

<apply>

<times/>

<ci> mwb537df2b_e6a2_4f13_a47d_d750a24d8eb2 </ci>

<ci> mwe70acfc0_34b6_4157_9905_e73cd58e595e </ci>

</apply>

<apply>

<times/>

<ci> mwc6ca25cc_7786_453b_90ab_a8c2ca24de0b </ci>

<ci> mwd9296a48_0796_4faa_87c8_e55eb6aa96bc </ci>

</apply>

</apply>

</math>

<listOfParameters>

<parameter id="mwb537df2b_e6a2_4f13_a47d_d750a24d8eb2" name="kin" value="0.00151" units="MWDERIVEDUNIT_1__second"/>

<parameter id="mwc6ca25cc_7786_453b_90ab_a8c2ca24de0b" name="kout" value="8.333e-05" units="MWDERIVEDUNIT_1__second"/>

</listOfParameters>

</kineticLaw>

</reaction>

<reaction id="mwb2093583_2e55_4f8e_85f3_b5b91447139a" name="degradation1" reversible="false" fast="false">

<listOfReactants>

<speciesReference species="mwe70acfc0_34b6_4157_9905_e73cd58e595e"/>

</listOfReactants>

<kineticLaw>

<math xmlns="http://www.w3.org/1998/Math/MathML">

<apply>

<times/>

<ci> mw5a958bbf_ba54_4d63_a327_12c0df5e5f82 </ci>

<ci> mwe70acfc0_34b6_4157_9905_e73cd58e595e </ci>

</apply>

</math>

<listOfParameters>

<parameter id="mw5a958bbf_ba54_4d63_a327_12c0df5e5f82" name="kdeg1" value="0.0004167" units="MWDERIVEDUNIT_1__second"/>

</listOfParameters>

</kineticLaw>

</reaction>

<reaction id="mw054b074a_8138_477d_84fc_8b9580a1a0e3" name="ARNT binding" fast="false">

<listOfReactants>

<speciesReference species="mwd9296a48_0796_4faa_87c8_e55eb6aa96bc"/>

<speciesReference species="mw4aba41df_adfc_4ccc_b5e3_7a838f8abed1"/>

</listOfReactants>

<listOfProducts>

<speciesReference species="mwf9e37e2b_58fb_42b3_ae54_34c6e14f0084"/>

</listOfProducts>

<kineticLaw>

<math xmlns="http://www.w3.org/1998/Math/MathML">

<apply>

<minus/>

<apply>

<times/>

<ci> mwcad16ce3_b239_4f14_83ce_a8d02ed227d9 </ci>

<ci> mwd9296a48_0796_4faa_87c8_e55eb6aa96bc </ci>

<ci> mw4aba41df_adfc_4ccc_b5e3_7a838f8abed1 </ci>

</apply>

<apply>

<times/>

<ci> mw8ecd9ff1_d8d4_48d2_b7f4_5bd0fac40a3f </ci>

<ci> mwf9e37e2b_58fb_42b3_ae54_34c6e14f0084 </ci>

</apply>

</apply>

</math>

<listOfParameters>

<parameter id="mwcad16ce3_b239_4f14_83ce_a8d02ed227d9" name="k_ARNT1" value="1.357e-07" units="MWDERIVEDUNIT_1__molecule_second"/>

<parameter id="mw8ecd9ff1_d8d4_48d2_b7f4_5bd0fac40a3f" name="k_ARNT2" value="8.333e-06" units="MWDERIVEDUNIT_1__second"/>

</listOfParameters>

</kineticLaw>

</reaction>

<reaction id="mw1b06da8b_87a9_40ef_96b3_578fd4fc6440" name="degradation2" reversible="false" fast="false">

<listOfReactants>

<speciesReference species="mwf9e37e2b_58fb_42b3_ae54_34c6e14f0084"/>

</listOfReactants>

<kineticLaw>

<math xmlns="http://www.w3.org/1998/Math/MathML">

<apply>

<times/>

<ci> mw2af56a4a_fe34_4dee_a6ce_0e928bb316ff </ci>

<ci> mwf9e37e2b_58fb_42b3_ae54_34c6e14f0084 </ci>

</apply>

</math>

<listOfParameters>

<parameter id="mw2af56a4a_fe34_4dee_a6ce_0e928bb316ff" name="kdeg2" value="0.00125" units="MWDERIVEDUNIT_1__second"/>

</listOfParameters>

</kineticLaw>

</reaction>

<reaction id="mw8315884b_c10b_4616_8f4b_0d2e193c7f1d" name="degradation3" reversible="false" fast="false">

<listOfReactants>

<speciesReference species="mwd9296a48_0796_4faa_87c8_e55eb6aa96bc"/>

</listOfReactants>

<kineticLaw>

<math xmlns="http://www.w3.org/1998/Math/MathML">

<apply>

<times/>

<ci> mw77cefcec_3584_4e21_8389_5813890c73be </ci>

<ci> mwd9296a48_0796_4faa_87c8_e55eb6aa96bc </ci>

</apply>

</math>

<listOfParameters>

<parameter id="mw77cefcec_3584_4e21_8389_5813890c73be" name="kdeg1" value="0.0004167" units="MWDERIVEDUNIT_1__second"/>

</listOfParameters>

</kineticLaw>

</reaction>

<reaction id="mw55c51c52_acfa_4ac6_b230_14ef79dbbec8" name="CoF binding" fast="false">

<listOfReactants>

<speciesReference species="mwf9e37e2b_58fb_42b3_ae54_34c6e14f0084"/>

<speciesReference species="mwfd381d44_fd59_4865_879d_f0676e15b3a2"/>

</listOfReactants>

<listOfProducts>

<speciesReference species="mw661625ec_78bf_4971_b983_e2b0553aa688"/>

</listOfProducts>

<kineticLaw>

<math xmlns="http://www.w3.org/1998/Math/MathML">

<apply>

<minus/>

<apply>

<times/>

<ci> mwf7ec7de3_d99f_47f4_8f39_912bfec3c181 </ci>

<ci> mwf9e37e2b_58fb_42b3_ae54_34c6e14f0084 </ci>

<ci> mwfd381d44_fd59_4865_879d_f0676e15b3a2 </ci>

</apply>

<apply>

<times/>

<ci> mw98d1460f_b982_4f37_860e_c3827920d1a7 </ci>

<ci> mw661625ec_78bf_4971_b983_e2b0553aa688 </ci>

</apply>

</apply>

</math>

<listOfParameters>

<parameter id="mwf7ec7de3_d99f_47f4_8f39_912bfec3c181" name="k_CoF_Abind" value="1.628e-06" units="MWDERIVEDUNIT_1__molecule_second"/>

<parameter id="mw98d1460f_b982_4f37_860e_c3827920d1a7" name="k_Cof_Aunbind" value="0.00025" units="MWDERIVEDUNIT_1__second"/>

</listOfParameters>

</kineticLaw>

</reaction>

<reaction id="mw3989160b_2cda_463a_af67_6343095b24f6" name="Other CoF binding" fast="false">

<listOfReactants>

<speciesReference species="mwfd381d44_fd59_4865_879d_f0676e15b3a2"/>

<speciesReference species="mw78afaec6_2a0b_4290_a12b_d967e9d89d31"/>

</listOfReactants>

<listOfProducts>

<speciesReference species="mwc646630d_524d_4b59_890b_fcdd19f0041f"/>

</listOfProducts>

<kineticLaw>

<math xmlns="http://www.w3.org/1998/Math/MathML">

<apply>

<minus/>

<apply>

<times/>

<ci> mwdd920f4f_b03a_4b4b_bcb1_d70a65469eae </ci>

<ci> mwfd381d44_fd59_4865_879d_f0676e15b3a2 </ci>

<ci> mw78afaec6_2a0b_4290_a12b_d967e9d89d31 </ci>

</apply>

<apply>

<times/>

<ci> mw121ba95d_7f63_4f5d_bd1f_1452c2940a6e </ci>

<ci> mwc646630d_524d_4b59_890b_fcdd19f0041f </ci>

</apply>

</apply>

</math>

<listOfParameters>

<parameter id="mwdd920f4f_b03a_4b4b_bcb1_d70a65469eae" name="k_CoF_Obind" value="1.628e-06" units="MWDERIVEDUNIT_1__molecule_second"/>

<parameter id="mw121ba95d_7f63_4f5d_bd1f_1452c2940a6e" name="k_CoF_Ounbind" value="0.00025" units="MWDERIVEDUNIT_1__second"/>

</listOfParameters>

</kineticLaw>

</reaction>

<reaction id="mw5e9202e9_a79a_4dfc_9935_eb191a723fed" name="AA_CoF DNA binding" fast="false">

<listOfReactants>

<speciesReference species="mw661625ec_78bf_4971_b983_e2b0553aa688"/>

<speciesReference species="mwd841ce40_b3fe_4388_b462_0e3b09be524d"/>

</listOfReactants>

<listOfProducts>

<speciesReference species="mw455a157e_ff7c_426b_a943_e1b8cd1cd808"/>

</listOfProducts>

<kineticLaw>

<math xmlns="http://www.w3.org/1998/Math/MathML">

<apply>

<minus/>

<apply>

<times/>

<ci> mw1fbd5366_b108_426f_8bfb_3af55a034b42 </ci>

<ci> mw661625ec_78bf_4971_b983_e2b0553aa688 </ci>

<ci> mwd841ce40_b3fe_4388_b462_0e3b09be524d </ci>

</apply>

<apply>

<times/>

<ci> mw71596603_3469_45d1_8810_2c702c5af147 </ci>

<ci> mw455a157e_ff7c_426b_a943_e1b8cd1cd808 </ci>

</apply>

</apply>

</math>

<listOfParameters>

<parameter id="mw1fbd5366_b108_426f_8bfb_3af55a034b42" name="k_CYPDRE_bind" value="4.33e-07" units="MWDERIVEDUNIT_1__molecule_second"/>

<parameter id="mw71596603_3469_45d1_8810_2c702c5af147" name="k_CYPDRE_unbind" value="0.00167" units="MWDERIVEDUNIT_1__second"/>

</listOfParameters>

</kineticLaw>

</reaction>

<reaction id="mw63b6ed1a_2070_4aaf_8f98_d84b984006af" name="DRE binding" fast="false">

<listOfReactants>

<speciesReference species="mw661625ec_78bf_4971_b983_e2b0553aa688"/>

<speciesReference species="mw9bea94b6_388a_46f0_9e30_8df1c202c739"/>

</listOfReactants>

<listOfProducts>

<speciesReference species="mw41e86661_220e_4030_8f3e_eba2564e78e3"/>

</listOfProducts>

<kineticLaw>

<math xmlns="http://www.w3.org/1998/Math/MathML">

<apply>

<minus/>

<apply>

<times/>

<ci> mw48cad4df_4f49_4e71_ae9f_332e23c81723 </ci>

<ci> mw661625ec_78bf_4971_b983_e2b0553aa688 </ci>

<ci> mw9bea94b6_388a_46f0_9e30_8df1c202c739 </ci>

</apply>

<apply>

<times/>

<ci> mw957de4a4_7757_411b_92b5_3b7d2fb43351 </ci>

<ci> mw41e86661_220e_4030_8f3e_eba2564e78e3 </ci>

</apply>

</apply>

</math>

<listOfParameters>

<parameter id="mw48cad4df_4f49_4e71_ae9f_332e23c81723" name="k_DRE_bind" value="1.628e-06" units="MWDERIVEDUNIT_1__molecule_second"/>

<parameter id="mw957de4a4_7757_411b_92b5_3b7d2fb43351" name="k_DRE_unbind" value="0.00167" units="MWDERIVEDUNIT_1__second"/>

</listOfParameters>

</kineticLaw>

</reaction>

<reaction id="mw531c732d_b60f_4d55_a2de_b9297dd5540f" name="CYP_Polbind" fast="false">

<listOfReactants>

<speciesReference species="mw455a157e_ff7c_426b_a943_e1b8cd1cd808"/>

<speciesReference species="mw1f0ddd1b_48a1_464a_951c_3b29d2b19572"/>

</listOfReactants>

<listOfProducts>

<speciesReference species="mw2973f81c_7b29_409d_b2ac_dc0be0780ad2"/>

</listOfProducts>

<kineticLaw>

<math xmlns="http://www.w3.org/1998/Math/MathML">

<apply>

<minus/>

<apply>

<times/>

<ci> mwe53db804_5a40_4e01_8f52_642f747f0b84 </ci>

<ci> mw455a157e_ff7c_426b_a943_e1b8cd1cd808 </ci>

<ci> mw1f0ddd1b_48a1_464a_951c_3b29d2b19572 </ci>

</apply>

<apply>

<times/>

<ci> mw88041a42_e9e2_44db_a890_f813978bad88 </ci>

<ci> mw2973f81c_7b29_409d_b2ac_dc0be0780ad2 </ci>

</apply>

</apply>

</math>

<listOfParameters>

<parameter id="mwe53db804_5a40_4e01_8f52_642f747f0b84" name="k_Polbind" value="0.4" units="MWDERIVEDUNIT_1__molecule_second"/>

<parameter id="mw88041a42_e9e2_44db_a890_f813978bad88" name="k_Polunbind" value="0.145" units="MWDERIVEDUNIT_1__second"/>

</listOfParameters>

</kineticLaw>

</reaction>

<reaction id="mwba5e4fd6_da9e_4313_b63d_df972b1b32c2" name="DRE_Polbind" fast="false">

<listOfReactants>

<speciesReference species="mw41e86661_220e_4030_8f3e_eba2564e78e3"/>

<speciesReference species="mw1f0ddd1b_48a1_464a_951c_3b29d2b19572"/>

</listOfReactants>

<listOfProducts>

<speciesReference species="mwec8f418e_d8a8_472e_8162_04976fca96ea"/>

</listOfProducts>

<kineticLaw>

<math xmlns="http://www.w3.org/1998/Math/MathML">

<apply>

<minus/>

<apply>

<times/>

<ci> mw270b989c_3a9b_47be_a013_3cadbba7f4fa </ci>

<ci> mw41e86661_220e_4030_8f3e_eba2564e78e3 </ci>

<ci> mw1f0ddd1b_48a1_464a_951c_3b29d2b19572 </ci>

</apply>

<apply>

<times/>

<ci> mw1586b76b_3c3c_47f7_9480_98c73234d42f </ci>

<ci> mwec8f418e_d8a8_472e_8162_04976fca96ea </ci>

</apply>

</apply>

</math>

<listOfParameters>

<parameter id="mw270b989c_3a9b_47be_a013_3cadbba7f4fa" name="k_DPolbind" value="0.4" units="MWDERIVEDUNIT_1__molecule_second"/>

<parameter id="mw1586b76b_3c3c_47f7_9480_98c73234d42f" name="k_DPolunbind" value="0.145" units="MWDERIVEDUNIT_1__second"/>

</listOfParameters>

</kineticLaw>

</reaction>

<reaction id="mw747d3f8c_4f82_4de7_81e4_6b611eed2f27" name="Initialization" reversible="false" fast="false">

<listOfReactants>

<speciesReference species="mw2973f81c_7b29_409d_b2ac_dc0be0780ad2"/>

</listOfReactants>

<listOfProducts>

<speciesReference species="mwf0ec6eb5_aaa2_47a8_a217_043de1df28f2"/>

</listOfProducts>

<kineticLaw>

<math xmlns="http://www.w3.org/1998/Math/MathML">

<apply>

<times/>

<ci> mw6deb47b8_859d_4018_9ae6_c118d6049867 </ci>

<ci> mw2973f81c_7b29_409d_b2ac_dc0be0780ad2 </ci>

</apply>

</math>

<listOfParameters>

<parameter id="mw6deb47b8_859d_4018_9ae6_c118d6049867" name="k_init" value="0.03" units="MWDERIVEDUNIT_1__second"/>

</listOfParameters>

</kineticLaw>

</reaction>

<reaction id="mw38e6be07_ab84_45f1_8e3b_61f584b0a1b0" name="ARNT_escape" fast="false">

<listOfReactants>

<speciesReference species="mwf0ec6eb5_aaa2_47a8_a217_043de1df28f2"/>

</listOfReactants>

<listOfProducts>

<speciesReference species="mw97f7ade5_32dd_455a_a70f_bd86fcf4b24e"/>

<speciesReference species="mw4aba41df_adfc_4ccc_b5e3_7a838f8abed1"/>

</listOfProducts>

<kineticLaw>

<math xmlns="http://www.w3.org/1998/Math/MathML">

<apply>

<minus/>

<apply>

<times/>

<ci> mw067a8cab_a611_4811_9f7c_e1fa1a6cd00a </ci>

<ci> mwf0ec6eb5_aaa2_47a8_a217_043de1df28f2 </ci>

</apply>

<apply>

<times/>

<ci> mw80bb3e6e_f5e3_4939_b0f1_a9ec9f8a39bc </ci>

<ci> mw97f7ade5_32dd_455a_a70f_bd86fcf4b24e </ci>

<ci> mw4aba41df_adfc_4ccc_b5e3_7a838f8abed1 </ci>

</apply>

</apply>

</math>

<listOfParameters>

<parameter id="mw067a8cab_a611_4811_9f7c_e1fa1a6cd00a" name="k_ARNT_off" value="0.005" units="MWDERIVEDUNIT_1__second"/>

<parameter id="mw80bb3e6e_f5e3_4939_b0f1_a9ec9f8a39bc" name="k_ARNT_backon" value="0.0055" units="MWDERIVEDUNIT_1__molecule_second"/>

</listOfParameters>

</kineticLaw>

</reaction>

<reaction id="mw7a425af2_be95_4c70_9969_0edec91bc63b" name="Termination" reversible="false" fast="false">

<listOfReactants>

<speciesReference species="mwf0ec6eb5_aaa2_47a8_a217_043de1df28f2"/>

</listOfReactants>

<listOfProducts>

<speciesReference species="mw1f0ddd1b_48a1_464a_951c_3b29d2b19572"/>

<speciesReference species="mwd841ce40_b3fe_4388_b462_0e3b09be524d"/>

<speciesReference species="mwf9e37e2b_58fb_42b3_ae54_34c6e14f0084"/>

<speciesReference species="mwfd381d44_fd59_4865_879d_f0676e15b3a2"/>

</listOfProducts>

<kineticLaw>

<math xmlns="http://www.w3.org/1998/Math/MathML">

<apply>

<times/>

<ci> mw8b3b6036_6041_4846_88c2_fe33324b0838 </ci>

<ci> mwf0ec6eb5_aaa2_47a8_a217_043de1df28f2 </ci>

</apply>

</math>

<listOfParameters>

<parameter id="mw8b3b6036_6041_4846_88c2_fe33324b0838" name="k_term" value="0.019" units="MWDERIVEDUNIT_1__second"/>

</listOfParameters>

</kineticLaw>

</reaction>

<reaction id="mw0d04f82d_d1ed_4b29_aa98_b500fc6a4c2e" name="Am_termination" reversible="false" fast="false">

<listOfReactants>

<speciesReference species="mw97f7ade5_32dd_455a_a70f_bd86fcf4b24e"/>

</listOfReactants>

<listOfProducts>

<speciesReference species="mw1f0ddd1b_48a1_464a_951c_3b29d2b19572"/>

<speciesReference species="mwfd381d44_fd59_4865_879d_f0676e15b3a2"/>

<speciesReference species="mwd9296a48_0796_4faa_87c8_e55eb6aa96bc"/>

</listOfProducts>

<kineticLaw>

<math xmlns="http://www.w3.org/1998/Math/MathML">

<apply>

<times/>

<ci> mw621946ba_c606_4470_accd_20e8a011d37a </ci>

<ci> mw97f7ade5_32dd_455a_a70f_bd86fcf4b24e </ci>

</apply>

</math>

<listOfParameters>

<parameter id="mw621946ba_c606_4470_accd_20e8a011d37a" name="k_Amterm" value="0.01" units="MWDERIVEDUNIT_1__second"/>

</listOfParameters>

</kineticLaw>

</reaction>

<reaction id="mw0d8ea994_afc3_40ef_a08d_8468a2a912a5" name="trans" reversible="false" fast="false">

<listOfReactants>

<speciesReference species="mwf0ec6eb5_aaa2_47a8_a217_043de1df28f2"/>

</listOfReactants>

<listOfProducts>

<speciesReference species="mwf105777a_6633_4581_b5f6_a44407454a24"/>

<speciesReference species="mwf0ec6eb5_aaa2_47a8_a217_043de1df28f2"/>

</listOfProducts>

<kineticLaw>

<math xmlns="http://www.w3.org/1998/Math/MathML">

<apply>

<times/>

<ci> mw64cb0a09_b599_422b_aa09_1633c09babdd </ci>

<ci> mwf0ec6eb5_aaa2_47a8_a217_043de1df28f2 </ci>

</apply>

</math>

<listOfParameters>

<parameter id="mw64cb0a09_b599_422b_aa09_1633c09babdd" name="k_trans" value="0.0013" units="MWDERIVEDUNIT_1__second"/>

</listOfParameters>

</kineticLaw>

</reaction>

<reaction id="mw72a4539b_bcea_42fe_8073_9e453577ce19" name="trans_Am" reversible="false" fast="false">

<listOfReactants>

<speciesReference species="mw97f7ade5_32dd_455a_a70f_bd86fcf4b24e"/>

</listOfReactants>

<listOfProducts>

<speciesReference species="mwf105777a_6633_4581_b5f6_a44407454a24"/>

<speciesReference species="mw97f7ade5_32dd_455a_a70f_bd86fcf4b24e"/>

</listOfProducts>

<kineticLaw>

<math xmlns="http://www.w3.org/1998/Math/MathML">

<apply>

<times/>

<ci> mwcc200e93_cf0a_4cef_8f04_2d3b955db1d9 </ci>

<ci> mw97f7ade5_32dd_455a_a70f_bd86fcf4b24e </ci>

</apply>

</math>

<listOfParameters>

<parameter id="mwcc200e93_cf0a_4cef_8f04_2d3b955db1d9" name="k_transAm" value="0.0008" units="MWDERIVEDUNIT_1__second"/>

</listOfParameters>

</kineticLaw>

</reaction>

<reaction id="mw13494cdb_5df7_4b15_953a_5c89ef5ef0af" name="mRNA_degradation" reversible="false" fast="false">

<listOfReactants>

<speciesReference species="mwf105777a_6633_4581_b5f6_a44407454a24"/>

</listOfReactants>

<kineticLaw>

<math xmlns="http://www.w3.org/1998/Math/MathML">

<apply>

<times/>

<ci> mw62f8e82d_bbbf_4e87_9961_d74950926b7d </ci>

<ci> mwf105777a_6633_4581_b5f6_a44407454a24 </ci>

</apply>

</math>

<listOfParameters>

<parameter id="mw62f8e82d_bbbf_4e87_9961_d74950926b7d" name="k_mRNA_deg" value="0.021" units="MWDERIVEDUNIT_1__second"/>

</listOfParameters>

</kineticLaw>

</reaction>

<reaction id="mwb2daa04e_f8fc_49ea_b35d_1bca04b20f68" name="Export" reversible="false" fast="false">

<listOfReactants>

<speciesReference species="mwf105777a_6633_4581_b5f6_a44407454a24"/>

</listOfReactants>

<listOfProducts>

<speciesReference species="mwae4b2487_0fa8_4a41_ad45_2ae11e66fdc5"/>

</listOfProducts>

<kineticLaw>

<math xmlns="http://www.w3.org/1998/Math/MathML">

<apply>

<times/>

<ci> mw1ee78119_40e0_476f_9827_6cdc2d020137 </ci>

<ci> mwf105777a_6633_4581_b5f6_a44407454a24 </ci>

</apply>

</math>

<listOfParameters>

<parameter id="mw1ee78119_40e0_476f_9827_6cdc2d020137" name="k_exp" value="0.002" units="MWDERIVEDUNIT_1__second"/>

</listOfParameters>

</kineticLaw>

</reaction>

<reaction id="mw2000340b_2a5d_4caa_8056_fe9acfa59b61" name="DRE_init" reversible="false" fast="false">

<listOfReactants>

<speciesReference species="mwec8f418e_d8a8_472e_8162_04976fca96ea"/>

</listOfReactants>

<listOfProducts>

<speciesReference species="mwdeca1e63_9abf_4025_90ce_0f9d548e2dc3"/>

</listOfProducts>

<kineticLaw>

<math xmlns="http://www.w3.org/1998/Math/MathML">

<apply>

<times/>

<ci> mw5d4f8dde_437a_4888_9a12_25dda7027729 </ci>

<ci> mwec8f418e_d8a8_472e_8162_04976fca96ea </ci>

</apply>

</math>

<listOfParameters>

<parameter id="mwd206ae07_65cd_4fcf_9c70_30a1c9c2ed42" name="k_Dinit" value="0.0001" units="MWDERIVEDUNIT_1__second"/>

<parameter id="mw5d4f8dde_437a_4888_9a12_25dda7027729" name="k_D_init" value="0.0001" units="MWDERIVEDUNIT_1__second"/>

</listOfParameters>

</kineticLaw>

</reaction>

<reaction id="mw136d3784_9478_44f4_88e7_64e346240799" name="D_termination" reversible="false" fast="false">

<listOfReactants>

<speciesReference species="mwdeca1e63_9abf_4025_90ce_0f9d548e2dc3"/>

</listOfReactants>

<listOfProducts>

<speciesReference species="mw9bea94b6_388a_46f0_9e30_8df1c202c739"/>

<speciesReference species="mwf9e37e2b_58fb_42b3_ae54_34c6e14f0084"/>

<speciesReference species="mwfd381d44_fd59_4865_879d_f0676e15b3a2"/>

<speciesReference species="mw1f0ddd1b_48a1_464a_951c_3b29d2b19572"/>

</listOfProducts>

<kineticLaw>

<math xmlns="http://www.w3.org/1998/Math/MathML">

<apply>

<times/>

<ci> mwf3de44d5_d8f8_42f2_a26d_69a70cd9e416 </ci>

<ci> mwdeca1e63_9abf_4025_90ce_0f9d548e2dc3 </ci>

</apply>

</math>

<listOfParameters>

<parameter id="mwf3de44d5_d8f8_42f2_a26d_69a70cd9e416" name="k_D_terminate" value="0.019" units="MWDERIVEDUNIT_1__second"/>

</listOfParameters>

</kineticLaw>

</reaction>

<reaction id="mw238ae459_30d4_400a_95f5_d04d486b6d2d" name="D_ARNT_escape" reversible="false" fast="false">

<listOfReactants>

<speciesReference species="mwdeca1e63_9abf_4025_90ce_0f9d548e2dc3"/>

</listOfReactants>

<listOfProducts>

<speciesReference species="mwe9d69afa_bb35_4453_bd01_c3db9dc6acea"/>

<speciesReference species="mw4aba41df_adfc_4ccc_b5e3_7a838f8abed1"/>

</listOfProducts>

<kineticLaw>

<math xmlns="http://www.w3.org/1998/Math/MathML">

<apply>

<times/>

<ci> mweccd5e80_331b_455a_9607_c0630cffa85e </ci>

<ci> mwdeca1e63_9abf_4025_90ce_0f9d548e2dc3 </ci>

</apply>

</math>

<listOfParameters>

<parameter id="mweccd5e80_331b_455a_9607_c0630cffa85e" name="k_DARNToff" value="0.005" units="MWDERIVEDUNIT_1__second"/>

</listOfParameters>

</kineticLaw>

</reaction>

<reaction id="mw6f6c8e1b_6649_45de_9792_d56c90b37534" name="D_Am_termination" reversible="false" fast="false">

<listOfReactants>

<speciesReference species="mwe9d69afa_bb35_4453_bd01_c3db9dc6acea"/>

</listOfReactants>

<listOfProducts>

<speciesReference species="mwd9296a48_0796_4faa_87c8_e55eb6aa96bc"/>

<speciesReference species="mw1f0ddd1b_48a1_464a_951c_3b29d2b19572"/>

<speciesReference species="mwfd381d44_fd59_4865_879d_f0676e15b3a2"/>

</listOfProducts>

<kineticLaw>

<math xmlns="http://www.w3.org/1998/Math/MathML">

<apply>

<times/>

<ci> mw051407ef_8ef7_4978_9603_1661f89b077d </ci>

<ci> mwe9d69afa_bb35_4453_bd01_c3db9dc6acea </ci>

</apply>

</math>

<listOfParameters>

<parameter id="mw051407ef_8ef7_4978_9603_1661f89b077d" name="k_DAm_terminate" value="0.01" units="MWDERIVEDUNIT_1__second"/>

</listOfParameters>

</kineticLaw>

</reaction>

<reaction id="mwbb0fb814_5946_4713_83ad_c35ae6c474d0" name="AHR_synthesis" reversible="false" fast="false">

<listOfProducts>

<speciesReference species="mwe0a75c34_5ffa_48fe_830f_bfcf5b6e61c5"/>

</listOfProducts>

<kineticLaw>

<math xmlns="http://www.w3.org/1998/Math/MathML">

<ci> mw8eaea1e8_9ad5_4cbf_a9b6_f57082cf96a6 </ci>

</math>

<listOfParameters>

<parameter id="mw8eaea1e8_9ad5_4cbf_a9b6_f57082cf96a6" name="k_AHRsynth" value="0.00142" units="MWDERIVEDUNIT_molecule__second"/>

</listOfParameters>

</kineticLaw>

</reaction>

<reaction id="mwb9deaadf_e8d3_4f5a_8e19_3fdb56468df4" name="ARNT_synthesis" reversible="false" fast="false">

<listOfProducts>

<speciesReference species="mw4aba41df_adfc_4ccc_b5e3_7a838f8abed1"/>

</listOfProducts>

<kineticLaw>

<math xmlns="http://www.w3.org/1998/Math/MathML">

<ci> mwe76b73d3_bdfb_49a5_a758_314206b0846b </ci>

</math>

<listOfParameters>

<parameter id="mwe76b73d3_bdfb_49a5_a758_314206b0846b" name="k_ARNTsynth" value="0.0013" units="MWDERIVEDUNIT_molecule__second"/>

</listOfParameters>

</kineticLaw>

</reaction>

<reaction id="mw88e05c39_d286_413a_94b7_2e0d5f071041" name="CoReg1_binding" fast="false">

<listOfReactants>

<speciesReference species="mw21412c31_04c5_44ef_9f26_8cf3ae3b1ddd"/>

<speciesReference species="mwf0ec6eb5_aaa2_47a8_a217_043de1df28f2"/>

</listOfReactants>

<listOfProducts>

<speciesReference species="mwf15006e7_b584_4348_90d9_9498e0fe36ac"/>

</listOfProducts>

<kineticLaw>

<math xmlns="http://www.w3.org/1998/Math/MathML">

<apply>

<minus/>

<apply>

<times/>

<ci> mw40f4d73d_695b_4c63_9cd4_99d8d1e28d83 </ci>

<ci> mw21412c31_04c5_44ef_9f26_8cf3ae3b1ddd </ci>

<ci> mwf0ec6eb5_aaa2_47a8_a217_043de1df28f2 </ci>

</apply>

<apply>

<times/>

<ci> mw9c98b1a1_540a_44b8_832c_03a8babdef29 </ci>

<ci> mwf15006e7_b584_4348_90d9_9498e0fe36ac </ci>

</apply>

</apply>

</math>

<listOfParameters>

<parameter id="mw40f4d73d_695b_4c63_9cd4_99d8d1e28d83" name="k_CR1on" value="0.0049" units="MWDERIVEDUNIT_1__molecule_second"/>

<parameter id="mw9c98b1a1_540a_44b8_832c_03a8babdef29" name="k_CR1off" value="0.005" units="MWDERIVEDUNIT_1__second"/>

</listOfParameters>

</kineticLaw>

</reaction>

<reaction id="mw380f995b_3f25_45aa_b7ef_62520c25342f" name="trans_CoR1" reversible="false" fast="false">

<listOfReactants>

<speciesReference species="mwf15006e7_b584_4348_90d9_9498e0fe36ac"/>

</listOfReactants>

<listOfProducts>

<speciesReference species="mwf105777a_6633_4581_b5f6_a44407454a24"/>

<speciesReference species="mwf15006e7_b584_4348_90d9_9498e0fe36ac"/>

</listOfProducts>

<kineticLaw>

<math xmlns="http://www.w3.org/1998/Math/MathML">

<apply>

<times/>

<ci> mw1da8a53d_442b_45ab_85a1_a17a059f60ac </ci>

<ci> mwf15006e7_b584_4348_90d9_9498e0fe36ac </ci>

</apply>

</math>

<listOfParameters>

<parameter id="mw1da8a53d_442b_45ab_85a1_a17a059f60ac" name="k_trans_CR1" value="0.0016" units="MWDERIVEDUNIT_1__second"/>

</listOfParameters>

</kineticLaw>

</reaction>

<reaction id="mw16036f2b_cc72_4a4c_83fc_3fa6200d6950" name="CR1_Am_binding" fast="false">

<listOfReactants>

<speciesReference species="mw97f7ade5_32dd_455a_a70f_bd86fcf4b24e"/>

<speciesReference species="mw21412c31_04c5_44ef_9f26_8cf3ae3b1ddd"/>

</listOfReactants>

<listOfProducts>

<speciesReference species="mw71c5a552_df36_4e17_87f2_3896da9028dc"/>

</listOfProducts>

<kineticLaw>

<math xmlns="http://www.w3.org/1998/Math/MathML">

<apply>

<minus/>

<apply>

<times/>

<ci> mw67d9d2ce_3f1d_469b_875a_c016db8e21c4 </ci>

<ci> mw97f7ade5_32dd_455a_a70f_bd86fcf4b24e </ci>

<ci> mw21412c31_04c5_44ef_9f26_8cf3ae3b1ddd </ci>

</apply>

<apply>

<times/>

<ci> mw3e61cad3_e65e_44b1_a6cf_67bbc8c49871 </ci>

<ci> mw71c5a552_df36_4e17_87f2_3896da9028dc </ci>

</apply>

</apply>

</math>

<listOfParameters>

<parameter id="mw67d9d2ce_3f1d_469b_875a_c016db8e21c4" name="k_Cr1Am_on" value="0.0049" units="MWDERIVEDUNIT_1__molecule_second"/>

<parameter id="mw3e61cad3_e65e_44b1_a6cf_67bbc8c49871" name="k_CR1Am_off" value="0.005" units="MWDERIVEDUNIT_1__second"/>

</listOfParameters>

</kineticLaw>

</reaction>

<reaction id="mw44c58f1e_a60d_4aa1_a29a_dcdc5a744e93" name="trans_Am_CR1" reversible="false" fast="false">

<listOfReactants>

<speciesReference species="mw71c5a552_df36_4e17_87f2_3896da9028dc"/>

</listOfReactants>

<listOfProducts>

<speciesReference species="mwf105777a_6633_4581_b5f6_a44407454a24"/>

<speciesReference species="mw71c5a552_df36_4e17_87f2_3896da9028dc"/>

</listOfProducts>

<kineticLaw>

<math xmlns="http://www.w3.org/1998/Math/MathML">

<apply>

<times/>

<ci> mw155d9673_0d6d_4570_82b0_6eb070945726 </ci>

<ci> mw71c5a552_df36_4e17_87f2_3896da9028dc </ci>

</apply>

</math>

<listOfParameters>

<parameter id="mw155d9673_0d6d_4570_82b0_6eb070945726" name="k_transAm_CR1" value="0.0011" units="MWDERIVEDUNIT_1__second"/>

</listOfParameters>

</kineticLaw>

</reaction>

<reaction id="mwb60007bd_1536_4446_8dd6_e6b0c9b88bca" name="CoR_bind" fast="false">

<listOfReactants>

<speciesReference species="mw21412c31_04c5_44ef_9f26_8cf3ae3b1ddd"/>

<speciesReference species="mwa662fddd_da79_454e_ab3d_4068859a29ba"/>

</listOfReactants>

<listOfProducts>

<speciesReference species="mw231f14b3_d84e_4d35_aac3_eaac7f318a3b"/>

</listOfProducts>

<kineticLaw>

<math xmlns="http://www.w3.org/1998/Math/MathML">

<apply>

<minus/>

<apply>

<times/>

<ci> mw822d971f_af72_4040_bcd6_3f97c7ef5fd0 </ci>

<ci> mw21412c31_04c5_44ef_9f26_8cf3ae3b1ddd </ci>

<ci> mwa662fddd_da79_454e_ab3d_4068859a29ba </ci>

</apply>

<apply>

<times/>

<ci> mw7112e73c_8dab_4c42_ba6b_eca9672bc69e </ci>

<ci> mw231f14b3_d84e_4d35_aac3_eaac7f318a3b </ci>

</apply>

</apply>

</math>

<listOfParameters>

<parameter id="mw822d971f_af72_4040_bcd6_3f97c7ef5fd0" name="kon_CoR" value="0.005" units="MWDERIVEDUNIT_1__molecule_second"/>

<parameter id="mw7112e73c_8dab_4c42_ba6b_eca9672bc69e" name="koff_CoR" value="0.0049" units="MWDERIVEDUNIT_1__second"/>

</listOfParameters>

</kineticLaw>

</reaction>

</listOfReactions>

</model>

</sbml>

**Table B. Reactions and Rate Constants**

| Reactions | | | | | | |
| --- | --- | --- | --- | --- | --- | --- |
| Reaction # | Description | Reversible | Forward rate | Backward rate | Forward equation | Backward equation |
| R1 | Diffusion of TCDD into cell | reversible | 8E-07 /s | 8E-07 /s | kf*[TCDD] | kb*[TCDD] |
| R2 | TCDD Binding to AHR | reversible | 5.3E-07 /(molecule-s) | 0.002 /s | Kf*[TCDD]*[AHR] | Kb*[AHR-TCDD] |
| R3 | Nuclear translocation | reversible | 1.51E-03 /s | 8.33E-05 /s | Kf*[AHR-TCDD]cyto | Kb*[AHR-TCDD]nuc |
| R4 | Cytoplasmic AHR degradation | Irrev. | 4.167E-04 /s |  | Kf*[AHR-TCDD]cyto | NA |
| R5 | ARNT binding | reversible | 1.357E-07 /(molecule-s) | 8.33E-06 /s | Kf*[AHR-TCDD]*[ARNT] | Kb*[AHR-ARNT] |
| R6 | ARNT-AHR complex degradation | Irrev. | 0.00125 /s | NA | K*[AHR-ARNT] | NA |
| R7 | Nuclear AHR degradation | Irrev. | 4.167E-04 /s | NA | K*[AHR-TCDD]nuc | NA |
| R8 | CoF binding | reversible | 1.628E-06 /(molecule-s) | 2.5E-04 /s | Kf*[AA]*[CoF] | Kb*[AA-CoF] |
| R9 | CoF binding to Other Sites | reversible | 1.628E-06 /(molecule-s) | 2.5E-04/s | Kf*[Other]*[CoF] | Kb*[Other-CoF] |
| R10 | AA-CoF binding to CYP1A1 DREs | reversible | 4.33E-07 /(molecule-s) | 0.00167 /s | Kf*[AA-CoF]*[CYPDRE] | Kb*[bound CYPDRE] |
| R11 | AACoF binding to Other DREs | reversible | 1.628E-06 /(molecule-s) | 0.00167 /s | Kf*[AA-CoF]*[DRE] | Kb*[bound DRE] |
| R12 | RNA Pol binding to CYP DRE complex | Reversible | 0.4 /(molecule-s) | 0.145 /s | Kf*[CYP-AA-CoF]*[RNAPol] | Kb*[CYP-AA-CoF-RNAPol] |
| R13 | RNA Pol binding to non-CYP DRE complex | Reversible | 0.4 /(molecule-s) | 0.145 /s | Kf*[DRE-AA-CoF]*[RNAPol] | Kb*[DRE-AA-CoF-RNAPol] |
| R14 | CYP Initialization | Irrev. | 0.03 / s |  | Kf*[CYP-AA-CoF-RNAPol] |  |
| R15 | ARNT escape | Reversible | 0.005 /s | 0.0055 / (molecule-s) | Kf*[CYP_init] | Kb*[CYP_init_Am][ARNT] |
| R16 | Termination | Irrev. | 0.019 /s |  | Kterm*CYP_init] |  |
| R17 | Termination | Irrev. | 0.01 /s |  | Kterm*[CYP_init_Am] |  |
| R18 | Transcription | Irrev. | 0.0013 /s |  | Ktrans*[CYP_init] |  |
| R19 | Transcription | Irrev | 0.0008 /s |  | Ktrans*[CYP_init_Am] |  |
| R20 | mRNA Degradation | Irrev. | 0.021 /s |  | Kdeg*[mRNA_t] |  |
| R21 | mRNA Export | Irrev. | 0.002 /s |  | Kexp*[mRNA_t] |  |
| R22 | DRE_init | Irrev. | 0.0001 /s |  | Kinit*[DRE-AA-CoF-RNAPol] |  |
| R23 | DRE termination | Irrev | 0.019 /s |  | Kterm*[DRE_init] |  |
| R24 | ARNT escape | Irrev. | 0.005 /s |  | Koff*[DRE_init] |  |
| R25 | DRE termination | Irrev. | 0.01 /s |  | Kterm*[DRE_init_Am] |  |
| R26 | AHR synthesis | Irrev. | 0.00142 /(molecule-s) |  | ksynth |  |
| R27 | ARNT synthesis | Irrev. | 0.0013 /(molecule-s) |  | ksynth |  |
| R28 | Coregulator binding to CYP | Reversible | 0.0049 /(molecule-s) | 0.005 /s | Kf*[CYP_init]*[CoReg] | Kb*[CYP_init-CoReg] |
| R29 | Transcription | Irrev. | 0.0016 /s |  | Ktrans*[CYP_init-CoReg] |  |
| R30 | Coregulator binding to CYP | Reversible | 0.0049 /s | 0.005 /s | Kf*[CYP_init_Am]*[CoReg] | Kb*[CYP_init_Am-CoReg] |
| R31 | Transcription | Irrev | 0.0011 /s |  | Ktrans*[CYP_init_Am-CoReg] |  |
| R32 | Coregulator competition | Reversible | 0.005 /(molecule-s) | 0.0049 /s | Kf*[CoReg]*[Comp] | Kb*[CoReg-Comp] |

**Table C. Comparison of classical rate constants, molecular rate constants and propensities**

| **Reaction #** | **Description** | **Classical Rate Constant** | **Molecular Rate Constant** |
| --- | --- | --- | --- |
| R3 | Binding to AHR (kf1) | 0.247 /(nmol/l – s) | 5.3E-07 /(molecule-s) |
| R8 | ARNT binding | 0.0025 /(nmol/l – s) | 1.357E-07 /(molecule-s) |
| R12 | CoF binding | 0.015 /(nmol/l – s) | 1.628E-06 /(molecule-s) |
| R14 | CoF binding to Others | 0.015 /(nmol/l – s) | 1.628E-06 /(molecule-s) |
| R16 | AA-CoF to CYP | 0.01 /(nmol/l – s) | 4.33E-07 /(molecule-s) |
| R18 | AACoF to DRE | 0.01 /(nmol/l – s) | 1.628E-06 /(molecule-s) |

**Table D. Hill Model fits of the Percentage of Cells with one or more CYP DRE bound**

| CoFactor | | Fitted Model Parameters | | |
| --- | --- | --- | --- | --- |
| Conc. (nM) | No. of Molecules | Hill Coefficient (n) | Half-maximal Concentration | Maximum |
| 0.065 | 20 | 1.283 | 1.392 | 27.6 |
| 0.195 | 60 | 1.273 | 1.888 | 66.88 |
| 0.651 | 200 | 1.300 | 0.9941 | 96.85 |
| 1.954 | 600 | 1.307 | 0.4207 | 100.4 |
| 3.256 | 1000 | 1.469 | 0.0645 | 100.0 |
| 6.512 | 2000 | 1.321 | 0.0195 | 101.2 |
| 9.768 | 3000 | 1.571 | 0.0200 | 101.0 |
| 19.54 | 6000 | 1.397 | 0.0201 | 101.2 |

**Equation A. Calculation of Percent Recruitment from the model results**

The equations below show the calculation for both AHR and ARNT using the species numbers from Supplementary Table 1 as subscripts.

$Percent Recruitment AHR=\frac{S_{13}+S_{16}+S_{19}+S_{20}+S_{25}+S_{26}}{S_{13}+S_{14}+S_{16}+S_{18}+S_{19}+S_{20}+S_{22}+S_{23}+S_{25}+S_{26}}$ Eq. S-1a

$Percent Recruitment ARNT=\frac{S_{13}+S_{16}+S_{19}+S_{25}}{S_{13}+S_{14}+S_{16}+S_{18}+S_{19}+S_{22}+S_{25}}$ Eq. S-1b

**Equation B. Calculation of Time-Averaged Numbers of ligand-bound AHR**

Using the interpolated average data over time obtained from the set of 100 runs, the following sum was calculated for each point in the time vector:

$ligand-bound AHR=S_{3}+S_{4}+S_{6}+S_{9}+S_{13}+S_{16}+S_{19}+S_{20}+S_{25}+S_{26}$ Eq. S-2

An average over time for this sum at was calculated and used as the response in Supplementary Figure S-1.

**Equation C. Calculation of Time-Averaged Numbers of cofactor bound to AHR-ARNT**

**$Bound Cofactor=S_{13}+S_{14}+S_{16}+S_{18}+S_{19}+S_{20}+S_{22}+S_{23}+S_{25}+S_{26}$** Eq. S-3

**Equation D. Equations used for Baseline Projection to determine Transitional Dose Values from Simon et al., (2014) [78]**

**$\begin{matrix} Response=\frac{1}{1+10^{n\left(\log_{10}\left(ED50\right)-\log_{10}\left(dose\right)\right)}} \\ \frac{d\left(Response\right)}{d\left(dose\right)}=Slope=\frac{n 10^{n\left(\log_{10}\left(ED50\right)-\log_{10}\left(dose\right)\right)}}{dose\left(1+10^{n\left(\log_{10}\left(ED50\right)-\log_{10}\left(dose\right)\right)}\right)} \\ \log_{10}\left(TDV\right)=\log_{10}\left(ED50\right)-\frac{0.21}{Slope} \end{matrix}$** Eq. S-4

**Figure A.** Dose-response to the number of ligand-bound AHR molecules as a function of TCDD concentration and fitted Hill functions. The Hill coefficient fit for all curves was 1.452 ± 0.1053 (best fit value ± std. error) and the EC50 was 0.3245 ± 0.01927 nM.


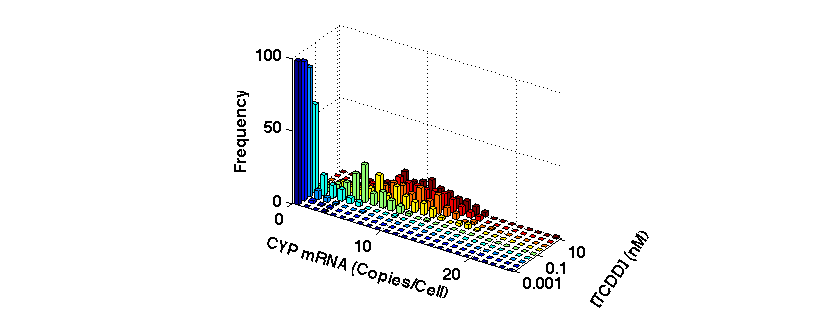


**Figure B.** Histograms of CYP1A1 mRNA copy number induced by TCDD

**Table E. Goodness of fit of Poisson distribution for modeled CYP1A1 mRNA copy number.**

| [TCDD] (nM) | Lambda (mean of Poisson distribution) | GOF (KS statistic and p-value) | Observed max # of mRNA copies (single cells) | Expected max # of mRNA copies (p99) | Observed Tissue # of mRNA copies (total for 100 cells) | Expected Range for Tissue # of mRNA copies (total for 100 cells) |
| --- | --- | --- | --- | --- | --- | --- |
| 0.001 | 0.05 | 0 (p = 0.8922) | 4 | 1 | 5 | 1 - 11 |
| 0.003 | 0.15 | 0 (p = 0.4402) | 4 | 2 | 15 | 7 - 25 |
| 0.01 | 0.41 | 0 (p = 0.4402) | 7 | 2 | 41 | 27 - 56 |
| 0.03 | 1.56 | 1, (p = 0.0308) | 10 | 5 | 156 | 129 - 186 |
| 0.1 | 6.07 | 1, (p = 0.0017) | 13 | 12 | 607 | 550 - 662 |
| 0.3 | 8.71 | 0 (p = 0.1383) | 18 | 16 | 871 | 802 - 940 |
| 1 | 10.07 | 1 (p = 0.2581) | 19 | 18 | 1007 | 928 - 1082 |
| 3 | 10.94 | 0 (p = 0.0680) | 17 | 19 | 1094 | 1018 - 1170 |
| 10 | 11.12 | 0 (p = 0.6738) | 25 | 20 | 1112 | 1038 - 1190 |
